# Alternating Shear Force of Respiration Regulates Cell Interactions of Fibroblasts and Macrophages to Promote Soft Tissue Integration of Chest Wall Polyetheretherketone Implants

**DOI:** 10.1002/advs.77118

**Published:** 2026-08-17

**Authors:** Rou Huang, Yangfan Huo, Jin Liu, Sida Liu, Minghai Ma, Hao Guo, Yuanquan Zhang, Xing Li, Xiao Liang, Jiawei Xiu, Ying Sun, Lijun Huang, Xiaolong Yan, Weiwei Wu, Lei Wang

**Affiliations:** ^1^ Department of Thoracic Surgery, Tangdu Hospital The Fourth Military Medical University Xi'an China; ^2^ School of Advanced Materials and Nanotechnology Xidian University Xi'an China; ^3^ Key Laboratory of Artificial Olfaction of Shaanxi Higher Education Institutes, School of Information Mechanics and Sensing Engineering Xidian University Xi'an China

**Keywords:** alternating shear force, cell interactions, soft tissue integration

## Abstract

Clinical studies have demonstrated that the infection rate at the incision site of polyetheretherketone (PEEK) implant in the craniomaxillofacial region is significantly higher than that in the chest wall. This difference is primarily attributable to the cyclic respiratory shear forces to which chest wall PEEK implants are subjected. However, the mechanisms by which cyclic respiratory shear force influences soft‐tissue integration around the PEEK interface have rarely been reported. Herein, we constructed an in vitro dynamic culture system to simulate the respiration‐induced shear force at the PEEK‐soft tissue interface. Our results showed that moderate alternating shear force promoted fibroblast proliferation and M2 macrophage polarization. Co‐culture experiments revealed that activated L929 fibroblasts induced M2 polarization of macrophages via paracrinally produced transforming growth factor beta 1 (TGF‐β1). Inversely, macrophages facilitated L929 fibroblast adhesion and migration by secreting TGF‐β1. Molecular tension probe measurements further demonstrated that integrin β1 (ITGB1) was activated at 12 pN. Animal experiments confirmed superior soft‐tissue integration around chest wall PEEK implants compared with craniomaxillofacial implants. These findings elucidate the core molecular mechanisms by which dynamic shear force mediates soft tissue integration at the PEEK‐tissue interface, thereby providing theoretical support for the clinical application of PEEK implants.

## Graphical abstract

Given that PEEK chest wall implants induce shear force at the soft tissue‐implant interface due to respiratory motion in the early in vivo post‐implantation stage. We confirmed that this mechanical stimulus triggers the PIEZO1/ITGB1/YAP1 signalling axis in L929 and the STAT6/p‐STAT6 pathway in RAW264.7, which in turn mediates adhesion and migration of L929 and M2 polarization of RAW264.7.

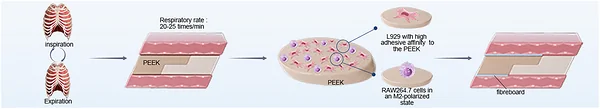

## 1 Introduction

Polyetheretherketone (PEEK) has emerged as a promising and applied polymeric implant material for bone defect reconstruction and clinical repair [1, 2, 3, 4], including those in the craniomaxillofacial region [5], spine [4, 6], oral cavity [7], and chest wall [8, 9]. However, PEEK has strong surface hydrophobicity and poor tissue affinity, which increases the risks of postoperative complications such as infection, implant loosening, and displacement [10]. A comparison of clinical studies on PEEK implants shows that the postoperative infection rate in the craniomaxillofacial region ranges from 10% to 32.8% [11, 12, 13], markedly higher than that in the chest wall (6.1%) [14, 15, 16]. Notably, these anatomical differences in complication rates are multifactorial and may arise from regional variations in blood supply, soft‐tissue thickness, and local microbial flora.

Beyond these conventional clinical factors, biomechanical discrepancies across anatomical sites represent another critical yet underappreciated variable. A further comparison of the implant environments between the chest wall and the craniomaxillofacial region reveals that, after chest wall implantation, PEEK is continuously subjected to respiratory motion, resulting in periodic micromotion between the implant and the residual bone as well as the surrounding soft tissues at a frequency of 20–40 times per minute [14, 17]. In contrast, PEEK implants placed in the craniomaxillofacial region and spine remain relatively stable and experience significantly less regular biomechanical stimulation [18]. Numerous studies have demonstrated that biomechanical stimulation can significantly regulate the regenerative repair process in soft tissues [19, 20, 21]. Xiao et al. demonstrated that cyclic mechanical stretching promotes wound healing by enhancing mitophagy and oxidative stress resistance in adipose‐derived stem cells via the PIEZO1/ATP axis [22]. Yuan et al. demonstrated that micromotion‐induced friction from microspheres effectively stimulates cellular activity, thereby promoting interfacial tissue fusion and regeneration [23]. Meanwhile, differences in the micro and nanotopography of the PEEK surface also markedly affect its integration with soft tissues. Our previous work further showed that PEEK interfaces with different stiffness can modulate the biological behavior of fibroblasts and macrophages, thereby improving soft tissue integration [24, 25]. Collectively, we hypothesize that site‐specific respiratory shear biomechanics serves as an independent regulatory factor that modulates PEEK interfacial integration, thereby partially accounting for the anatomical discrepancy in clinical complication rates. Nevertheless, how sustained, regular respiratory shear forces regulate soft tissue integration around the PEEK interface remains poorly understood, and the underlying molecular mechanisms await further elucidation.

Herein, we established an in vitro bionic platform [26], namely a dynamic cell culture system, engineered to recapitulate the physiological mechanical loads that implants endure in the human body during the early post‐implantation phase. This system was further leveraged to dissect the cellular responses and tissue integration mechanisms at the implant–soft tissue interface under dynamic mechanical stimulation, focusing on fibroblasts and macrophages as the two principal cell types orchestrating PEEK implant–soft tissue integration [25, 27, 28]. Given that PEEK chest wall implants induce shear force at the soft tissue‐implant interface due to respiratory motion in the early in vivo post‐implantation stage (Scheme 1A), we applied cyclic shear stress to L929 fibroblasts and RAW264.7 macrophages seeded on PEEK substrates. We confirmed that this mechanical stimulus triggers the PIEZO1/ITGB1/YAP1 signalling axis in L929 and the STAT6/p‐STAT6 pathway in RAW264.7, which in turn mediates adhesion and migration of L929 and M2 polarization of RAW264.7. Furthermore, molecular tension probes measurements revealed for the first time that integrin β1is activated under a mechanical tension of 12 pN (Scheme 1B). This study clarifies the key regulatory role of respiratory alternating shear force in promoting soft tissue integration around PEEK implants and reveals the mechanism by which specific biomechanical signals affect the biological performance of bone implants. These findings provide a theoretical basis for the design of PEEK implants tailored to different anatomical sites. The clinical implication is that moderate biomechanical stimulation at the implant interface can effectively promote tissue healing and integration. Future work may adopt similar mechanical regulation strategies, such as physical interventions including extracorporeal ultrasound, to optimize the implant microenvironment and further improve the clinical outcomes of PEEK implants.

**SCHEME 1:**
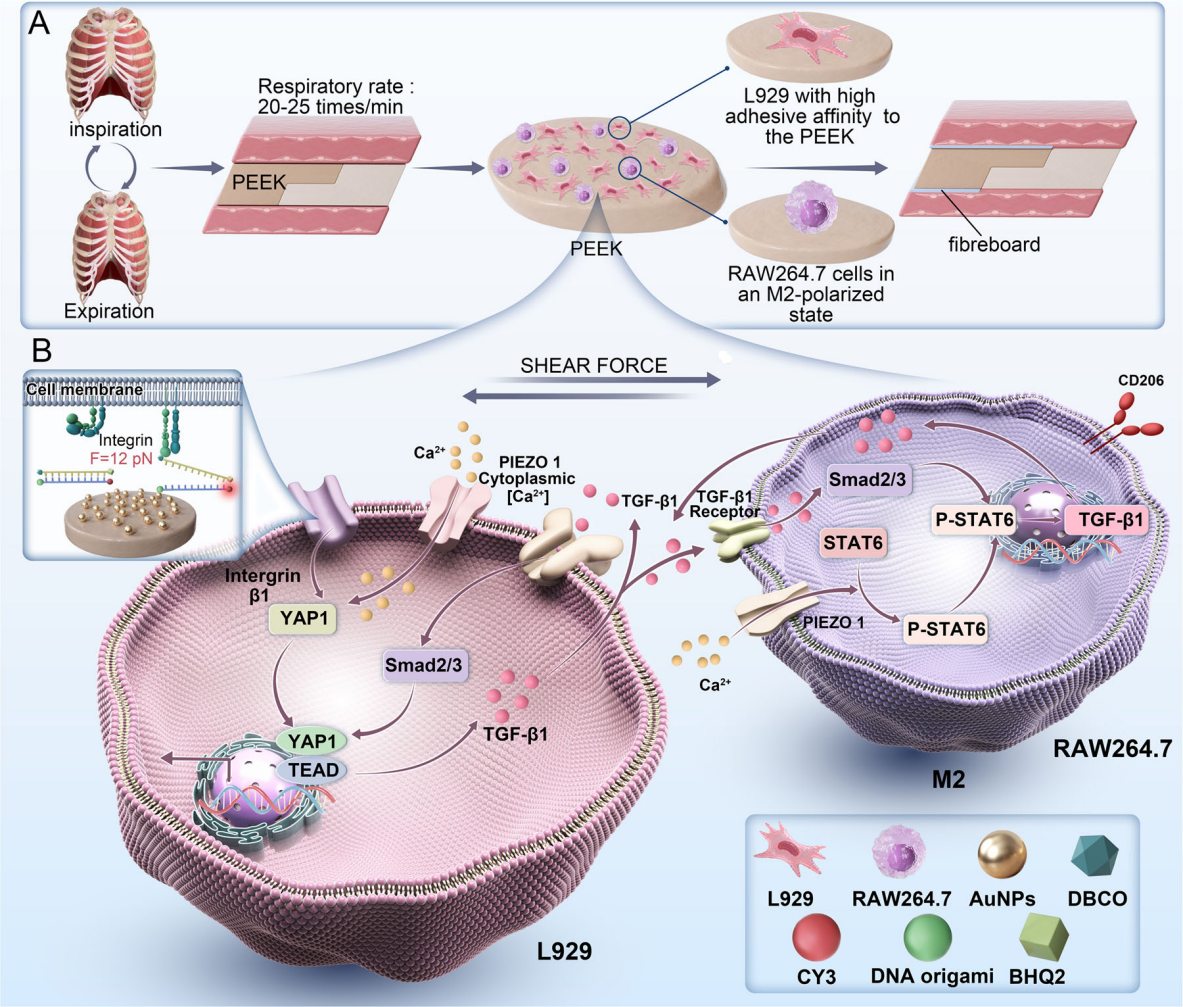
Schematic diagram. (A) The interface between the PEEK implant and surrounding soft tissue is subjected to alternating shear stress under physiological respiratory motion. (B) Alternating shear stress mediates the activation of the PIEZO1/ITGB1/YAP1 pathway in fibroblasts and the STAT6/p‐STAT6 pathway in RAW264.7 macrophages.

## 2 Results

### 2.1 Characterization of PEEK Chest Wall Implants and Finite Element Simulation of Physiological Shear Force in the Dynamic Culture System

PEEK was prepared by 3D printing into a circular disk with a diameter of 1 cm and a thickness of 1 mm. Macroscopic imaging (Figure 1A) confirmed the smooth, featureless surface of the as‐prepared PEEK disk. Further, the microstructure and elemental composition of the material were characterized using scanning electron microscopy (SEM) and energy‐dispersive X‐ray spectroscopy (EDS). As shown in Figure 1B, the PEEK surface was relatively smooth, with carbon (C) and oxygen (O) uniformly distributed across it. Surface roughness was is directly related to the friction coefficient, which impacted the integration of soft tissue and the implant interface in chest wall implants. The surface morphology and roughness of the PEEK disk, as characterized by atomic force microscopy (AFM) (Figure 1C), showed a roughness value of 0.0702 µm, which was within a range conducive to protein adsorption, cell spreading, and tissue integration [29]. In addition, we employed X‐ray photoelectron spectroscopy (XPS) to investigate the chemical groups and elemental composition of PEEK (Figure 1D). The results indicated that the PEEK surface was composed solely of C and O; no extraneous elemental signals were detected, demonstrating high chemical purity of the PEEK surface. The surface hardness of the PEEK was measured via nanoindentation to be approximately 3.8 GPa (Figure 1E). This value falls within the range of human cortical bone hardness (approximately 3–20 GPa) [30], indicating its potential to provide adequate mechanical support in chest wall construction.

**FIGURE 1:**
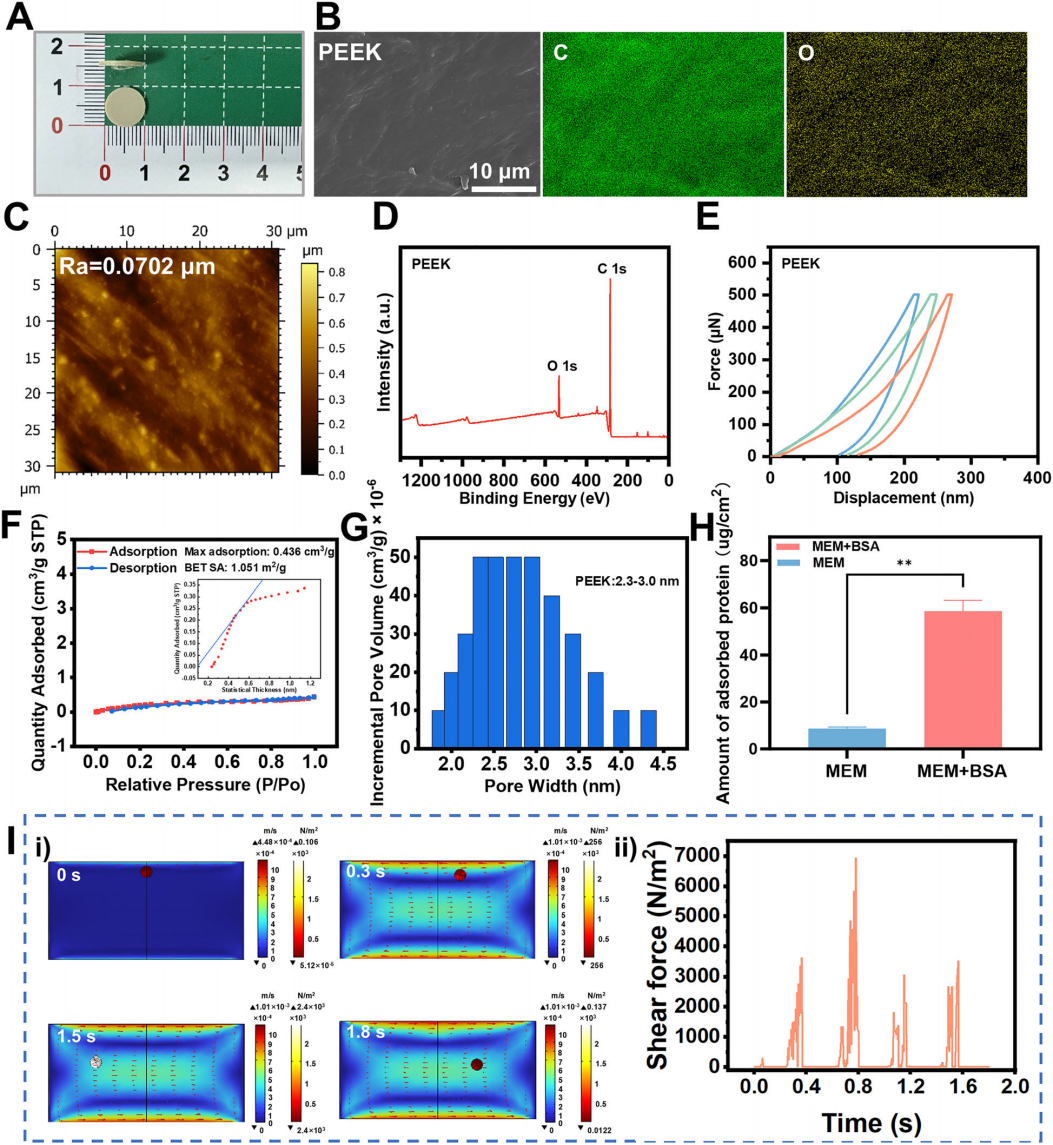
Characterization of PEEK implants and cellular mechanical stimulation modeling. (A) PEEK disc photos. (B) SEM and (C) AFM images of PEEK. (D) XPS energy spectrum analysis of PEEK. (E) Load‐displacement curve of PEEK. (F) Nitrogen adsorption–desorption isotherms of PEEK. (G) Pore size distribution curve of PEEK. (H) Quantitative analysis of static BSA adsorption amount. (I) i)Finite element simulation and analysis of mechanical forces on a single cell in dynamic cell culture, ii) Von Mises stress time history curve.

We further assessed its surface porosity and static protein adsorption, two key parameters governing implant–tissue interfacial performance. The nitrogen adsorption–desorption data showed that the PEEK surface was dense and essentially pore‐free (Figure 1F,G). This structural characteristic renders the material unlikely to be invaded by irregular substances or to undergo uncontrolled tissue overgrowth, which, in turn, would favor a more predictable pattern of soft‐tissue integration around the implant. For protein binding, BSA was used as the model protein at a concentration of 4.5 mg/mL. The adsorption rate we obtained was 1.39%, indicating that nonspecific protein adhesion on PEEK is relatively limited (Figure 1H). Given that excessive protein fouling has been associated with aggravated inflammatory reactions, this low level of deposition, when coupled with the dense surface texture, may help moderate the early foreign body response. And if the initial inflammatory phase is contained, one might expect the subsequent tissue remodeling process to proceed in a more orderly fashion.

To verify that our established dynamic cell culture system can accurately recapitulate the cyclic shear force generated by physiological respiratory motion at the PEEK‐soft tissue interface in vivo, we performed fluid‐structure interaction (FSI) finite element simulation using COMSOL Multiphysics. The simulation results demonstrated that, under the specified dynamic cell culture parameters, the cell layer on the implant surface was subjected to cyclically varying fluid shear force (Figure 1I‐i). The stress waveform exhibited an approximately sinusoidal pattern with a period of approximately 1.8 s, corresponding to a frequency of approximately 0.56 Hz, which aligns with the physiological frequency of normal breathing [31]. Figure 1I‑ii presents the von Mises equivalent stress–time history curve for a single cell within the dynamic cell culture system. The results indicated that theinterfacial stress peaks ranged from 3 to 7 kPa. Specifically, the stress interval of 3–5 kPa falls within the physiological stress range of normal human respiration (0.1–5 kPa) [32], whereas the peak at 7 kPa corresponds to stress levels associated with extreme physiological loading, such as deep breathing. These findings further confirm that the dynamic culture model we developed can effectively recapitulate the biomechanical microenvironment generated by human respiratory movements at the implant‐tissue interface.

### 2.2 Shear Force Stimulation Induced L929 Cell Proliferation, Adhesion and Migration

Fibroblasts are core effector cells in soft tissue regeneration and remodelling. They synthesize and secrete extracellular matrix (ECM) components, cytokines, and growth factors. These substances maintain tissue structural homeostasis and functional integrity. Dysregulated fibroblast activity links to pathological conditions. Examples include excessive tissue fibrosis and tumour progression. We compared the biological behaviors (proliferation, adhesion, migration) of L929 cells cultured on PEEK substrates under dynamic mechanical stimulation (mimicking respiratory shear force) versus static conditions, to clarify how physiological mechanical cues regulate fibroblast function during PEEK implant integration. As shown in Figure S1, the cell proliferation of L929 on the PEEK surface was continuously monitored for 5 days following mechanical stimulation. A significant difference in cell viability appeared at 12 h post‐mechanical stimulation (Figure 2A). During the first three days, dynamic stimulation significantly promoted proliferation (*P < 0.01*) compared to the static group. The proliferative advantage diminished in the next two days (*P < 0.05*). This was primarily attributed to contact inhibition induced by increased cell confluency. Meanwhile, colony formation assays were conducted on L929 cells cultured under static and dynamic conditions. As shown in Figure S2, the number of cell colonies in the dynamic culture group was significantly higher than that in the static group. Cell adhesion is the initial step for implant‐tissue integration. We tracked the dynamic adhesion process of L929 cells on PEEK for the first 6 h using confocal fluorescence microscopy (nuclei stained with DAPI, cytoplasm stained with FITC‐phalloidin) (Figure 2B and Figure S3). Within the first 2 h, cells in both groups were spherical. Many of them were suspended in the supernatant. By 4 h, dynamic group cells had fully spread on PEEK. Static group cells remained incompletely spread. A small number of suspended cells still existed. At 6 h, no suspended cells were detected in either group. All cells achieved complete adhesion. This showed dynamic stimulation accelerated L929 adhesion on PEEK. At 12 h, cell viability and adhesion were further assessed using live/dead staining (Figure 2C). No significant cell death was observed in the dynamic group, with a higher number of green‐fluorescent live cells relative to the static control. These results not only confirm the excellent biocompatibility of the dynamic culture system but also provide indirect evidence that shear force promotes cell adhesion and proliferation.

**FIGURE 2:**
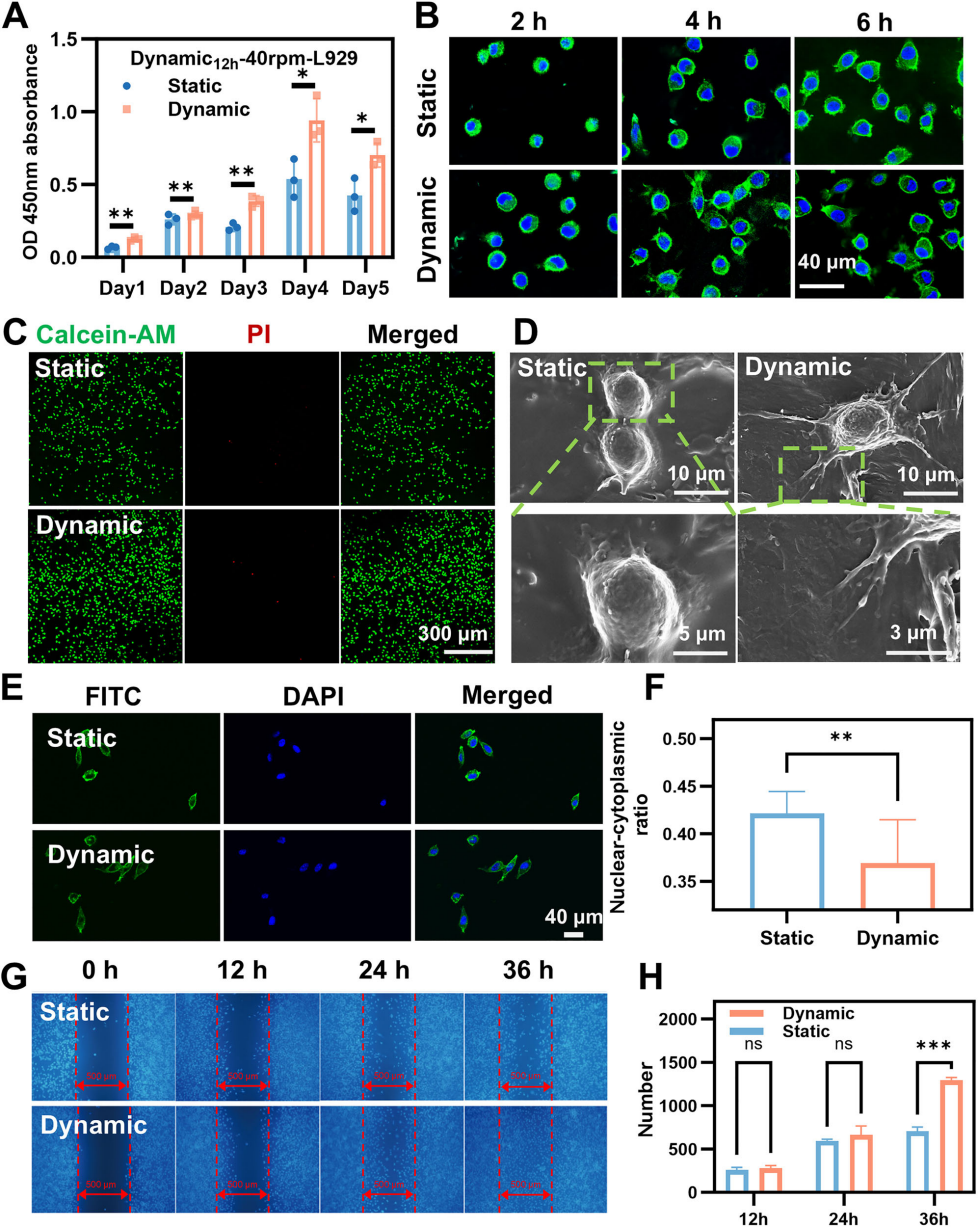
Biological behaviors of L929 on PEEK surfaces under different culture conditions. (A) L929 cells were cultured under dynamic (40 rpm) and static conditions for 12 h, and cell viability was measured by CCK‐8 assay. (B) Fluorescence images of L929 at 2, 4, and 6 h of dynamic and static culture. (C) Live/dead staining of L929 fibroblasts after 12 h of culture (green: live cells; red: dead cells). (D) SEM images of L929 cultured on PEEK for 12 h. (E) Confocal images of L929 (nucleus: DAPI, blue; cytoskeleton: FITC‐phalloidin, green) after 12 h of culture (scale bar: 40 µm). (F) Quantitative analysis of the nucleocytoplasmic (N/C) ratio in L929 cells. (G) Wound healing assays of L929 under different culture conditions. (H) Quantitative analysis of the wound healing rate (n = 3; ^***^ *P* < 0.001). Shown are mean values ± SD (n = 3 independent experiments, each with 3 technical replicates), ^*^ *P* < 0.05, ^**^ *P* < 0.01, and ^***^ *P* < 0.001, ns, no significant difference. 2‐way ANOVA was used in (A) and (H), and an unpaired *t*‐test was used in (F).

The morphology of L929 cells on PEEK after 12 h under static or dynamic conditions was characterized by SEM. As shown in Figure 2D, statically cultured cells maintained a hemispherical shape without extending distinct filopodia or lamellipodia. In contrast, dynamically stimulated cells displayed prominent lamellipodia, a more spread morphology, and tighter adhesion to PEEK. This phenotypic shift suggests that shear force enhances the mechano‐cellular response, promoting optimal adhesion of L929 to the PEEK surface.

In addition to morphological alterations, cell adhesion was evaluated based on the fluorescence intensity ratio of DAPI (nuclear stain) to FITC (cytoskeletal stain). Representative images and quantitative analysis were presented in Figure 2E, respectively. L929 on PEEK under dynamic culture conditions exhibited a greater area of green fluorescence compared to those under static conditions. Quantitative analysis of the nucleus‐to‐cytoplasm (DAPI/FITC) fluorescence intensity ratio revealed that the value for L929 cells in the dynamic group (0.3692 ± 0.04559) was significantly lower than that in the static group (0.4214 ± 0.02328) (Figure 2F). This reduction indicates that mechanical stimulation effectively promotes L929 adhesion and spreading, which can be attributed to greater cytoplasmic spreading relative to nuclear size in response to these cues.

Cell migration is a key biological process that mediates soft tissue repair and the integration of implant‐tissue interfaces. The migratory capacity of L929 was evaluated using a standardized scratch assay (initial wound width: 500 µm) (Figure 2G). Due to the opacity of PEEK, cell nuclei in the scratch area were stained with DAPI and visualized by fluorescence microscopy at different time points. Cells in both groups gradually migrated into the cell‐free scratch area over time; however, statistical analysis of the number of migrated cells revealed a significant difference between the two groups at 36 h post‐scratch (*P < 0.001*) (Figure 2H). The dynamic group exhibited a substantially higher number of migrated cells compared to the static group, indicating that cyclic shear stress enhances the migratory capacity of L929 on PEEK surfaces. This enhanced cellular migration facilitates the formation of a stable soft tissue interface surrounding PEEK.

### 2.3 Shear Force Mediates Integration of Soft Tissue by Activating PIEZO1/ITGB1/YAP1 in L929

Dynamic mechanical stimulation promotes the proliferation, adhesion, and migration of L929 on PEEK surfaces, characteristics that facilitate implant‐tissue integration. To elucidate the potential mechanotransduction pathways driving these responses and soft tissue integration, further molecular investigations were conducted (Figure 3A). GO enrichment analysis showed significant enrichment for processes including cell adhesion, extracellular matrix organization, and responses to external stimuli and fluid shear force (Figure 3B). Consistent with this, KEGG pathway analysis identified enrichment in biomechanically relevant pathways, including ECM‐receptor interaction and fluid shear stress‐related pathways (Figure 3C). Using RT‐qPCR, the mRNA expression levels in L929 cells in response to mechanical stimulation were detected. The results showed that after 12 h of mechanical stimulation, the transcription levels of gene sets related to adhesion and migration were all increased (Figure S4). These findings collectively imply that subsequent research should focus on cytoskeletal dynamic remodeling and mechanotransduction pathways in L929 following mechanical stimulation.

**FIGURE 3:**
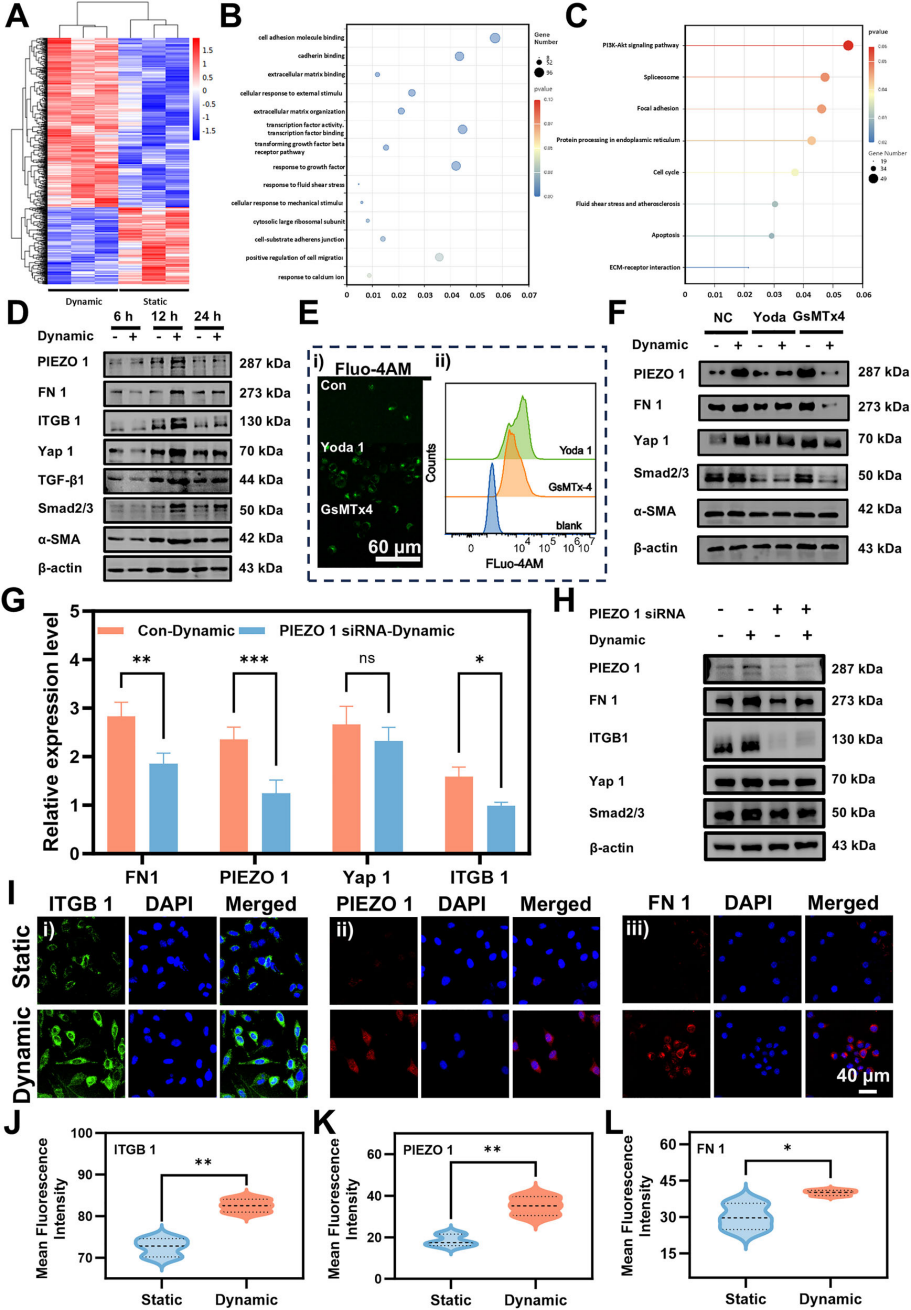
Mechanical force regulates gene and protein expression in L929 cells. (A) Heatmap of differentially expressed genes in L929 cells cultured on PEEK surfaces under dynamic vs. static conditions. (B) GO enrichment analysis and (C) KEGG pathway enrichment analysis of differentially expressed genes. (D) Time‐dependent expression of mechanosensing, proliferation, and fibrosis‐related proteins in dynamically cultured L929 cells, determined by Western blot. (E) Changes in cell fibrosis and proliferation‐related proteins after activation and inhibition of PIEZO1 protein function. (i) Confocal fluorescence images showed the accumulation of calcium in L929, (ii) Flow cytometry was used to evaluate the effect of PIEZO1 on calcium influx in L929. (F) The regulation of PIEZO1 on adhesion‐related proteins was evaluated on PEEK at 12 h of dynamic culture. (G) Knockdown of PIEZO1 via siRNA in L929 cells abrogated the upregulation of PIEZO1, ITGB1, and FN1 expression triggered by dynamic respiratory shear stress. (H) Knockdown of PIEZO1 by siRNA in L929 cells suppressed the PIEZO1/ITGB1/YAP1 axis activation. (I) Confocal fluorescence images of ITGB1, PIEZO1, and FN1 in L929 under dynamic and static conditions (scale bar: 40 µm). The average fluorescence intensity of (J) ITGB1, (K) PIEZO1, and (L) FN1 was statistically analyzed. Shown are mean values ± SD (n = 3 independent experiments, each with 3 technical replicates), ^*^ *P* < 0.05, ^**^ *P* < 0.01, and ^***^ *P* < 0.001, ns, no significant difference. 2‐way ANOVA was used in (G, J—L).

As a pivotal mechanosensitive ion channel, PIEZO1 mediates cellular responses to mechanical stimuli via conformational changes that open its Ca^2^^+^‐permeable channel, triggering rapid intracellular Ca^2^^+^ influx. This signal served as a second messenger to activate downstream molecules including yes‐associated protein 1 (YAP1) and integrin β1 (ITGB1). Based on the convergence of this canonical mechanism and our transcriptomic findings suggesting mechano‐sensitive pathway activation, we examined the protein expression of these targets by Western blotting. PIEZO1 upregulation showed a positive temporal correlation with YAP1 activation and increased ITGB1 abundance (Figure 3D). This indicates that force‐induced PIEZO1 accumulation potentially augments YAP1 and integrin adhesion signaling through prolonged Ca^2^^+^ influx, thus providing the upstream molecular impetus for the time‐dependent enhancement of cell spreading and adhesion. Importantly, the time‐dependent enhancement in cell spreading and adhesion, driven by force‐induced PIEZO1/YAP1/ITGB1 signaling, remodels the extracellular matrix (ECM) microenvironment to further sustain and amplify mechanical force‐triggered upregulation of TGF‐β1/Smad2/3. Increased cell adhesion promotes ECM deposition (such as collagen, fibronectin) and cross‐linking, which in turn activates latent TGF‐β1 sequestered in the ECM via latent TGF‐β1 binding proteins. The released active TGF‐β1 ligands bind to their receptors, creating a persistent mechanical‐chemical feedback loop that sustains Smad2/3 activation. Collectively, this process is initiated by shear force, transduced via the PIEZO1/YAP1/ITGB1/Ca^2^^+^ axis, and amplified by YAP1‐Smad2/3 crosstalk, integrin signaling, and ECM remodeling. Furthermore, α‐smooth muscle actin (α‐SMA) and fibronectin 1 (FN1) are core marker proteins for the occurrence and progression of fibrosis, with their expression levels markedly upregulated upon mechanical stimulation. The upregulation of α‐SMA induces the transdifferentiation of fibroblasts into myofibroblasts, which in turn secrete abundant FN1. Excessive FN1 deposition triggers ECM remodelling, thereby further driving the remodelling of PEEK‐surrounding soft tissues.

To confirm the role of PIEZO1 in Ca^2^^+^ influx and downstream fibrosis, the inhibitor Grammostola spatulata mechanotoxin 4 (GsMTx4) and activator Yoda1 were employed. Confocal imaging with Fluo‐4AM revealed that PIEZO1 activation elevated intracellular Ca^2^^+^ levels in L929 cells, whereas GsMTx4 abolished mechanical stimulation‐induced Ca^2^^+^ accumulation (Figure 3E‐i), a finding corroborated by flow cytometry (Figure 3E‐ii). Subsequent experiments demonstrated that modulation of PIEZO1 by GsMTx4 and Yoda1 regulated FN1 synthesis (Figure 3F). Consistently, siRNA‐mediated knockdown of PIEZO1 significantly suppressed the expression of FN1, ITGB1, and PIEZO1 induced by dynamic shear force (Figure 3G), and attenuated the activation of the PIEZO1/ITGB1/YAP1 axis (Figure 3H). The observation that mechanical stimulation failed to elevate ITGB1 expression upon PIEZO1 depletion strongly indicates that intact PIEZO1 function is an indispensable upstream prerequisite for force‐mediated ITGB1 upregulation, which is executed via a signaling axis wherein PIEZO1‐gated Ca^2^^+^ entry activates Calpain to cleave Talin1, yielding its bioactive form that accelerates ITGB1 aggregation and activation for ECM remodeling. The consequent ECM stiffening further reinforces PIEZO1 mechanosensing through an outside‐in feedback loop [26, 33].

As a key early ECM scaffold protein, PIEZO1‐dependent FN1 expression directly contributes to aberrant ECM deposition and assembly, thereby initiating mechanical force‐induced soft tissue remodelling. Immunofluorescence staining revealed that mechanical stimulation significantly upregulated ITGB1 and FN1 expression, both of which accumulated at the L929 cell membrane, suggesting early ECM remodelling (Figure 3I‐i, iii). Quantitative fluorescence intensity analysis confirmed significantly higher levels of ITGB1 and FN1 in the dynamic group than in the static group (both *P < 0.01*), further validating the regulatory effect of shear force (Figure 3J–L). Notably, PIEZO1 exhibited marked accumulation near the nuclear membrane in dynamically cultured cells (Figure 3I‐ii), with quantification showing a statistically significant difference compared to static controls (*P < 0.001)* (Figure 3K). These findings suggest that mechanical force activates the PIEZO1/ITGB1/YAP1 axis, contributing to the initiation of soft tissue fibrosis and bridging cellular responses with molecular mechanisms.

### 2.4 Quantitative Analysis of Integrin Mechanotransduction at the PEEK Interface Using DNA Origami Tension Probes

Our previous work identified the PIEZO1/ITGB1/YAP1 axis as a central mediator of mechanical force‐induced fibroblast activation and early soft tissue integration around PEEK implants. Integrin β1, as a key mechanosensor and upstream component of this signalling cascade, serves as the initial point of contact for translating extracellular mechanical cues into intracellular biochemical signals. To quantitatively map the piconewton (pN)‐scale forces governing integrin β1 activation at the PEEK interface, we designed a DNA origami‐based tension probe to visualize and quantify integrin‐mediated forces at the PEEK‐cell interface. As schematically illustrated in Figure 4A, this modular platform consists of three core components: a PEEK substrate functionalized with gold nanoparticles (AuNPs) (Figure S5), a DNA origami nanostructure acting as a force‐sensitive mechanical spring [34], and an azide‐modified integrin ligand (K(N_3_)GRGDNP) that selectively engages integrin β1. The DNA origami is site‐specifically anchored to the AuNPs via thiol‐gold chemistry, while the integrin ligand is conjugated to the distal end of the DNA duplex by a DBCO‐azide click reaction. A Förster resonance energy transfer (FRET) pair, consisting of a Cy3 fluorophore and a BHQ2 quencher, is integrated into the DNA duplex onto the square origami scaffold by DNA hybridization. The probe operates on a tension‐dependent fracture mechanism: under low mechanical tension, the DNA duplex remains intact, maintaining FRET and quenching Cy3 fluorescence; when cellular traction exceeds the predefined threshold, strand dissociation physically separates the fluorophore from the quencher, activating a fluorescent readout. By engineering probes with distinct force thresholds, we converted invisible piconewton‐scale mechanical signals into detectable outputs, enabling direct visualization of integrin‐mediated mechanotransduction. To systematically delineate the mechanical activation landscape of fibroblasts, we established three tiered force thresholds of 12, 33, and 56 pN based on established mechanobiological benchmarks [34]: 10–20 pN for initiating ITGB1‐mediated mechanosensing, 30–40 pN for driving focal adhesion maturation [35], and forces exceeding 50 pN for inducing supraphysiological stress responses [36]. This graded design enabled us to pinpoint the specific traction regime engaged by cells under dynamic mechanical loading.

**FIGURE 4:**
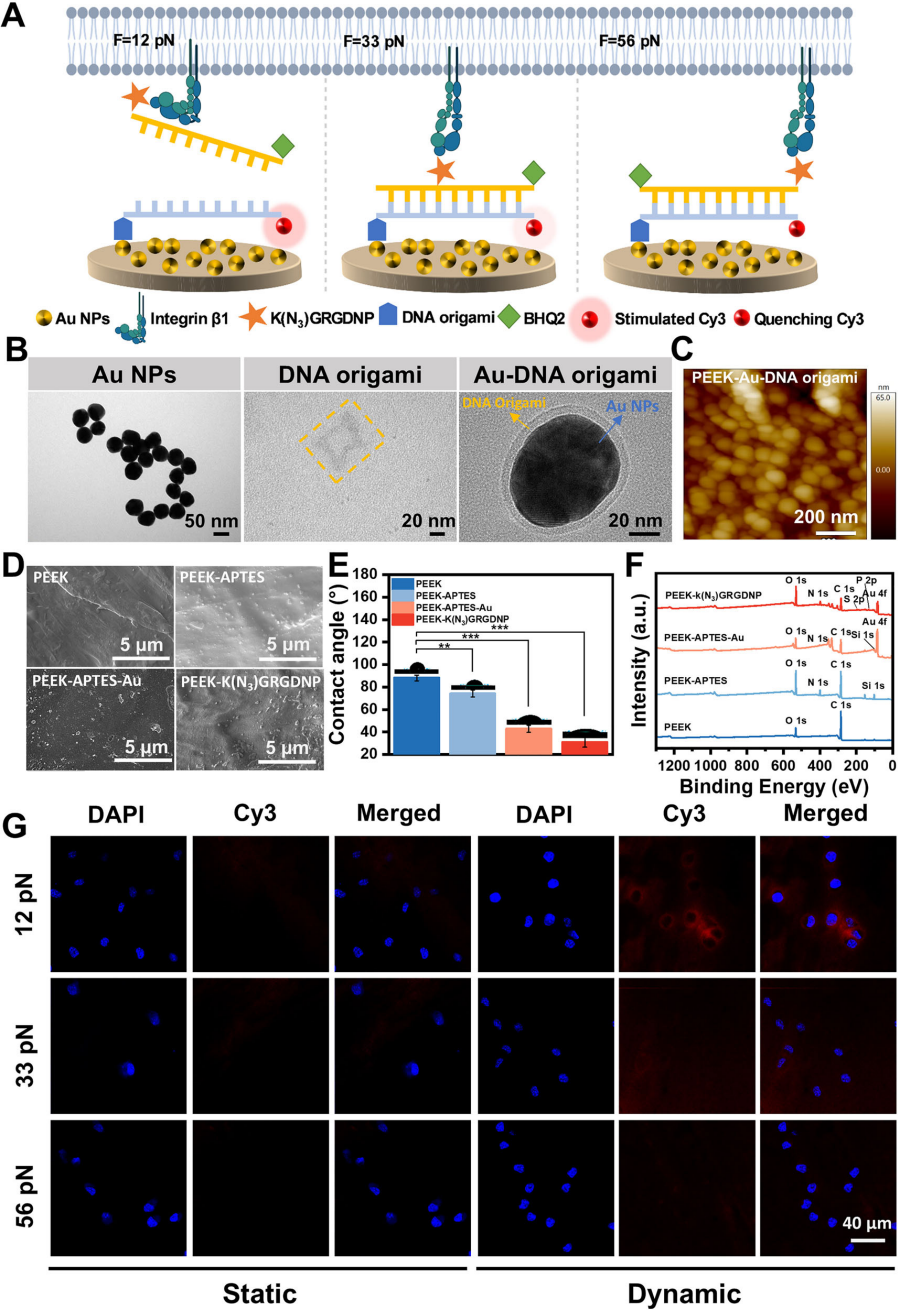
Characterization of PEEK surface integrin tension sensor and mapping of integrin tension during cell adhesion. (A) Schematic representation of L929 pulling on the varied threshold of rupture probe surfaces 12, 33, and 56 pN. (B) TEM images of bare Au nanoparticles (AuNPs, scale bar: 50 nm), pristine DNA origami nanostructures (scale bar: 100 nm), and Au‐DNA origami hybrid complex (scale bar = 20 nm). (C) AFM images of PEEK‐Au‐DNA origami. (D) SEM images of PEEK, PEEK‐APTES, PEEK‐APTES‐Au, and PEEK‐K(N3)GRGDNP. (E) Hydrophilicity of different sample groups was measured using a water contact angle measuring instrument. (F) XPS energy spectrum analysis of the modified PEEK samples. (G) Confocal fluorescence images (Cy3: red, DAPI: blue) showing tension‐dependent probe activation under static vs. dynamic culture conditions (scale bar: 40 µm).

To confirm intact probe assembly. Stepwise TEM verified uniform AuNPs, intact folded DNA origami templates, and complete core‐shell composite structures without fragmentation (Figure 4B). EDS elemental co‐localization of Au, P, and S further confirmed tight encapsulation of Au cores by DNA origami shells (Figure S6A). AFM was employed to characterize morphological alterations during stepwise functionalization of the probes on the PEEK surface (Figure S6B). Following 3‐aminopropyltriethoxysilane (APTES) treatment, the PEEK surface was converted from a relatively smooth morphology to a rougher, pitted texture, providing increased anchoring sites and elevated specific surface area for subsequent AuNPs immobilization. To ensure sufficient probe density for effective cell adhesion and spreading, we calibrated AuNPs distribution, revealing an average interparticle spacing of ∼70 nm, which satisfies the minimal density requirement for reliable probe presentation. A polyacrylamide gel image indicates that the DBCO‐modified DNA strand was successfully modified with the K(N_3_)GRGDNP integrin ligand (Figure S7). AFM imaging further revealed that individual DNA origami monomers displayed a well‐defined square‐like morphology (Figures S8 and S9). However, when the DNA origami was grafted onto the AuNPs‐decorated PEEK surface, we were unable to clearly resolve its structural features using AFM. This limitation arises primarily because the thickness of the DNA origami scaffold is substantially smaller than the inherent topographical roughness of the underlying PEEK substrate, which masks the subtle morphological variations of the nanostructures. Our combined TEM analysis demonstrates that DNA origami was successfully anchored onto the PEEK surface via AuNPs serving as bridging connectors (Figure 4C). Relative to irregular assemblies, this uniform square configuration facilitates more balanced intermolecular hydrogen‐bonding interactions, thereby conferring enhanced mechanical stability and structural integrity to the DNA origami scaffold. This structural feature minimized nonspecific deformation or rupture under physiological mechanical tension (from several pN to tens of pN) exerted by L929 cells, ensuring that DNA duplex dissociation occurs only upon reaching the predefined tension threshold and thus improving the accuracy of mechanosensing measurements.

The morphological evolution of the modified PEEK surfaces was further visualized using SEM (Figure 4D). Water contact angle measurements confirmed that sequential functionalization with APTES, AuNPs, and DNA origami progressively enhanced surface hydrophilicity. The improved wettability promoted a uniform distribution of AuNPs, creating a more favorable interface for DNA origami immobilization (Figure 4E). XPS validated the stepwise surface functionalization. The initial APTES modification was confirmed by the emergence of a characteristic N1s signal, indicating successful amination. Subsequent AuNPs deposition induced a marked reduction in N1s intensity, consistent with efficient grafting via Au–N coordination bonds that alter the local electronic environment or partially shield amine groups. Following immobilization of the DNA‑origami–peptide complex, the N1s peak was restored, accompanied by the appearance of a distinct P2p peak and a weak S2p signal. The phosphorus signal arises from the phosphate backbone of DNA origami, while the sulfur signal originates from thiol groups mediating the spontaneous adsorption of DNA structures onto AuNPs. The nitrogen signal can be attributed to both the azide moiety in the K(N_3_)GRGDNP ligand and the nucleobases of DNA. This sequential evolution of characteristic elemental signals unambiguously verifies the successful fabrication pathway: APTES amination of PEEK, AuNPs anchoring, and final immobilization of the DNA‑origami–peptide probe complex (Figure 4F). DNA origami‑based molecular tension probes with distinct force thresholds (12, 33, and 56 pN) were fabricated on the PEEK surface. L929 cells were cultured on these probe‑modified surfaces under static or dynamic mechanical conditions for 12 h, and fluorescence signals were monitored by confocal laser scanning microscopy. Under static conditions, no obvious fluorescence activation was observed around cell membranes for any of the three tension thresholds. This indicates that the forces generated by L929 cells via focal adhesions on static PEEK‑DNA substrates were generally below 12 pN, insufficient to rupture the probe. In contrast, under dynamic culture, robust red fluorescence was detected at the cell periphery on the 12 pN probe surfaces, weak red fluorescence was detected around the cells on the surface of the 33 pN probe, whereas no fluorescence was observed on the 56 pN probes (Figure 4G). These findings confirm that dynamic shear stress elevates cellular traction within the physiological 12 pN matching native fibroblast adhesion mechanics, without generating supraphysiological mechanical overload, consistent with the physiological force range governing integrin–fibronectin interactions.

In summary, using DNA origami tension probes, we quantitatively determined that dynamic mechanical stimulation drives L929 cells to exert traction forces of 12 pN on the substrate via activated integrins. This physiological 12 pN tension acts as the upstream mechanical trigger of the PIEZO1/ITGB1/YAP1 cascade, initiating ITGB1 activation, PIEZO1‐mediated Ca^2^^+^ influx, YAP1 nuclear translocation, and TGF‐β1‐dependent ECM remodeling to facilitate PEEK soft tissue integration. Partial 33 pN probe response and absent 56 pN signal further demonstrate that this pro‐integrative phenotype depends specifically on moderate threshold‐level integrin tension, rather than excessive or pathological mechanical force. These findings establish a direct mechanistic link whereby the dynamic respiratory‐like mechanical microenvironment promotes soft tissue integration around PEEK implants.

### 2.5 Shear Force Stimulation Mediates Macrophage M2 Polarization on PEEK Surfaces via a PIEZO1‐STAT6/NF‐κB Crosstalk Axis

Macrophages serve as central regulators of soft tissue remodelling around implants. Owing to their high phenotypic plasticity, they play a critical regulatory role in the transition from the inflammatory foreign body reaction to reparative tissue integration. These cells exhibit dynamic switching between pro‐inflammatory M1 and pro‐reparative M2 subtypes. M1 macrophages initiate early tissue clearance and inflammatory responses, whereas M2 macrophages resolve inflammation, secrete pro‐reparative cytokines, and modulate fibroblast activation. Given that PEEK chest wall implants are continuously subjected to cyclic shear force generated by physiological respiratory movements, we propose that this endogenous dynamic mechanical microenvironment directly regulates cell behavior, thereby reshaping the peri‐implant immune microenvironment and promoting soft tissue integration. In various types of cells, cyclic shear force acts as a vital mechanobiological signal that is sensed by the macrophage mechanosensitive PIEZO1 channel, mostly triggering Ca^2^^+^ influx and initiating downstream signaling cascades. This mechanical signal exerts a time‐dependent regulatory effect on macrophage polarization by differentially tuning NF‐κB and STAT6 signaling activity. To elucidate the regulatory effects of mechanical cues on macrophage polarization, we first analyzed the expression profiles of proteins associated with macrophage phenotypic switching and functional regulation under mechanical stimulation at different time points using Western Blot. Our results showed that mechanical stimulation for 6 h activated PIEZO1, inducing Ca^2^^+^ influx and subsequent activation of YAP1 and inducible nitric oxide synthase (iNOS), while suppressing CD206 expression, indicating a pronounced M1 polarization of macrophages. With prolonged cyclic shear loading, continuous PIEZO1 activation gradually suppresses the pro‐inflammatory NF‐κB pathway and preferentially activates the STAT6‐dependent reparative program, driving phenotypic transformation toward M2 macrophages. With the extension of mechanical stimulation duration, the expression of STAT6/p‐STAT6, TGF‐β1, and CD206 was significantly upregulated, whereas the NF‐κB/p‐NF‐κB signaling pathway was inhibited. Collectively, these findings demonstrate that early shear force stimulation drives the polarization of macrophages toward the pro‐inflammatory M1 phenotype by activating the PIEZO1/Ca^2^^+^/YAP1 axis, and sustained mechanical stimulation initiates an endogenous feedback regulatory mechanism that switches macrophage polarization from the M1 to the pro‐reparative M2 phenotype via STAT6 activation and NF‐κB inhibition (Figure 5A).

**FIGURE 5:**
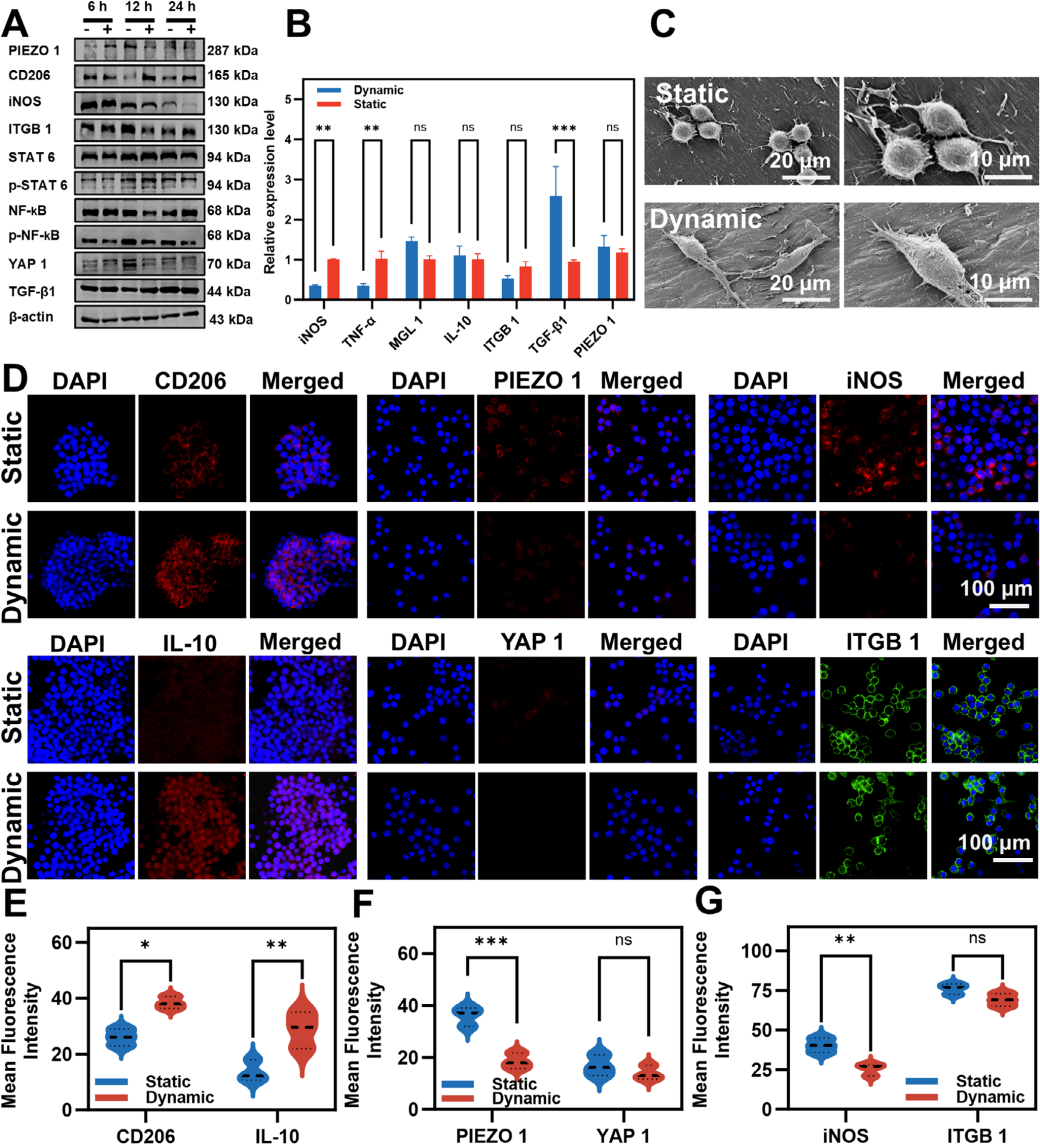
Mechanical force modulates macrophage M2 polarization on PEEK surfaces. (A) The expression levels of mechanical perception (PIEZO1, YAP1) and polarization‐related proteins (iNOS, CD206, STAT6/p‐STAT6, NF‐κB/p‐NF‐κB) in dynamically cultured RAW264.7, determined by Western blot. (B) Gene expression in RAW264.7 were cultured under dynamic and static conditions for 12 h. (C) SEM images of RAW264.7 under static and dynamic culture conditions (scale bar: 10 µm). (D) Confocal fluorescence images of CD206, PIEZO1, iNOS, IL10, YAP1 and ITGB1 in macrophages under dynamic and static conditions (scale bar: 100 µm). The average fluorescence intensity of (E) CD206, IL‐10, (F) PIEZO1, YAP1, and (G) iNOS, ITGB1 were statistically analyzed. Shown are mean values ± SD (n = 3 independent experiments, each with 3 technical replicates), ^*^ *P* < 0.05, ^**^ *P* < 0.01, and ^***^ *P* < 0.001, ns, no significant difference. 2‐way ANOVA was used in (B) and (E–G).

Based on the time‐course analysis by western blotting, 12 h was selected as the time point for subsequent functional and phenotypic characterization. RT‐qPCR was performed to detect the changes in mRNA transcription levels of polarization‐related markers in RAW264.7 cells under mechanical stimulation. The results indicated that mechanical stimulation led to a significant downregulation of M1 polarization‐associated markers iNOS and tumor necrosis factor‐alpha (TNF‐α) (*P < 0.01*), while the expression of the M2 polarization‐related marker TGF‐β1 was significantly increased (*P < 0.001*) (Figure 5B). To rigorously define the immune phenotype elicited by dynamic shear force, we incorporated canonical positive control groups for both M1 and M2 polarization. Specifically, RAW264.7 macrophages were stimulated with lipopolysaccharide (LPS) to induce a pro‐inflammatory M1 state, or with a combination of interleukin‐4 (IL‐4) and interleukin‐10 (IL‐10) to promote an anti‐inflammatory M2 phenotype, serving as reference benchmarks (Figure S10). Corroborating the transcriptional alterations described above, immunoblotting analyses revealed that dynamic mechanical stimulation markedly upregulated the M2‐associated markers CD206 and phosphorylated STAT6 (p‐STAT6), while concurrently attenuating NF‐κB phosphorylation (p‐NF‐κB). Notably, the molecular signature imparted by shear force bore a striking resemblance to the anti‐inflammatory profile of IL‐4/IL‐10–treated M2 macrophages, and stood in sharp contrast to the pro‐inflammatory program elicited by LPS challenge. Collectively, these findings substantiate the regulatory role of mechanical forces in steering macrophage polarization. To further verify the essential role of PIEZO1 in mediating mechanically regulated macrophage phenotypic transition, we knocked down PIEZO1 expression via siRNA transfection under dynamic shear culture conditions (Figure S11). Consistent with the above mechanistic results, knockdown of PIEZO1 increased total STAT6 protein expression and downregulated NF‐κB protein abundance. These results suggest that PIEZO1 is a critical upstream mechanosensor that modulates macrophage responses to mechanical stimulation, which is consistent with the reports in the literature [37].

Furthermore, we explored the effect of 12 h mechanical force stimulation on RAW264.7 cells on the PEEK surface. In the control group, under static conditions, RAW264.7 cells on the PEEK surface exhibited a circular shape with filopodia extending from the cells. In contrast, the morphology of cells after mechanical force stimulation changed significantly, showing a spindle shape with both filopodia and lamellipodia around the cells, and enhanced cell polarity—morphological changes that are consistent with the M2 polarization tendency observed in the WB and RT‐qPCR results (Figure 5C). Similarly, immunofluorescence staining confirmed that after 12 h of mechanical force stimulation, the expression levels of the M2 polarization‐related proteins CD206 and IL‐10 in RAW264.7 cells were significantly increased, while the expression of the M1 polarization‐related protein iNOS was significantly decreased (Figure 5D). The analysis results of average fluorescence intensity further verified the above trends (Figure 5E–G).

Collectively, these results reveal a novel time‐dependent mechanotransduction pathway in RAW264.7, in which cyclic shear force induced by respiratory motion activates PIEZO1/Ca^2^^+^ signalling. This signalling cascade sequentially regulates YAP1, STAT6, and NF‐κB, thereby driving a functional M1‐to‐M2 phenotypic transition in RAW264.7 cells, with 12 h as a critical time window for this reparative conversion. This mechanical regulatory mechanism of macrophage polarization complements our previously observed PIEZO1/ITGB1/YAP1‐mediated L929 activation, together establishing a coordinated cellular response pattern to physiological mechanical signals at the PEEK–soft tissue interface.

### 2.6 Shear Force Stimulation Drives the Interplay Between RAW264.7 and L929 by Remodeling Their Paracrine Microenvironment

Fibroblast‐macrophage communication, primarily mediated by paracrine signalling, coordinates the transition from inflammation to tissue repair. Given that dynamic mechanical stimulation independently activates PIEZO1/ITGB1/YAP1 signalling in L929 fibroblasts and induces M2 polarization of RAW264.7 macrophages, we hypothesized that physiological mechanical cues further modulate their paracrine output, forming a pro‐reparative regulatory loop that enhances PEEK implant integration. Here, we employed an indirect co‐culture system using conditioned media (CM) to dissect the mechanical regulation of this intercellular crosstalk (Figure 6A). Based on the optimal 12 h time point identified in our monoculture studies, L929 and RAW264.7 were cultured separately under either dynamic or static conditions for 12 h. After the culture completion, the culture supernatants were collected and centrifuged to remove cellular debris, and the resulting supernatant was defined as CM. Subsequently, these conditioned media were mixed at specified volumetric ratios (e.g., L929‐CM: RAW264.7‐CM = 1:3, 1:1, 3:1) to create a gradient in paracrine signal intensity. This experimental design was employed to evaluate the functional impact of macrophage‐derived paracrine signals on fibroblast activity. Specifically, we first examined the proliferative response of L929 fibroblasts treated with CM derived from RAW264.7 macrophages cultured under static or dynamic mechanical stimulation. Cell proliferation was quantitatively assessed using the CCK‐8 assay kit on days 1, 2, 3, 4, and 5 post‐treatment. Notably, on day 3, the CM mixed at a 1:1 ratio induced the most significant proliferative response among the groups (*P < 0.01*). While the 1:1 mixed CM from dynamically stimulated macrophages robustly promoted L929 cell proliferation (Figure 6B), both the group with a higher concentration of RAW264.7‐CM (1:3) and the group with a lower concentration of RAW264.7‐CM (3:1) failed to elicit substantial changes compared to the control group (Figure 6C,D). Therefore, the 1:1 ratio was selected for all subsequent functional and mechanistic studies.

**FIGURE 6:**
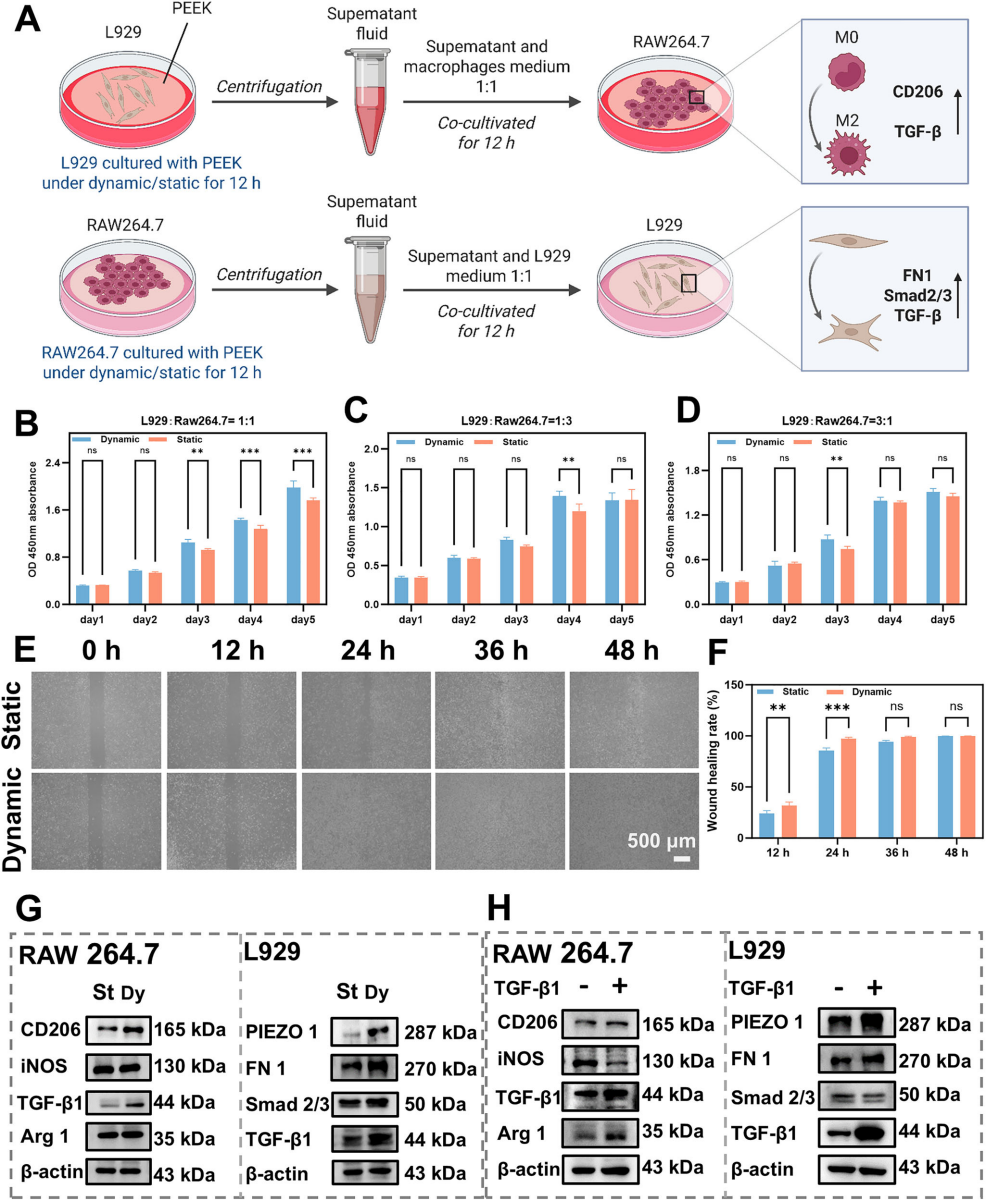
Mechanical stimulation drives the interplay between RAW264.7 and L929 by remodeling their paracrine microenvironment. (A) Schematic of the indirect co‐culture system using CM from L929 and RAW264.7. Effect of paracrine on L929 at (B) 1:1, (C) 1:3, and (D) 3:1 ratio of L929:RAW264.7, cell viability was measured by CCK‐8 assay. (E) Representative scratch assay images of L929 treated with dynamic RAW264.7‐CM, static RAW264.7‐CM(scale bar: 500 µm). (F) Quantitative analysis of wound closure rate. (G) Changes in the expression of characteristic proteins in L929 and RAW264.7 cells after indirect co‐culture. (H) Effect of TGF‐β1 on the expression of related proteins in L929 and RAW264.7. Shown are mean values ± SD (n = 3 independent experiments, each with 3 technical replicates), ^*^ *P* < 0.05, ^**^ *P* < 0.01, and ^***^ *P* < 0.001, ns, no significant difference. 2‐way ANOVA was used in (B–D) and (F).

Building upon the observed pro‐proliferative effect, we investigated whether the paracrine factors present in macrophage‐conditioned media could also influence L929 fibroblast migration. Using a standardized scratch assay (initial wound width: 500 µm), we quantified cell migration into the denuded area over 48 h. Representative images (Figure 6E) clearly show that L929 cells treated with Dynamic RAW264.7‐CM (1:1) migrated more rapidly to close the wound compared to those treated with static RAW264.7‐CM (1:1). Quantitative analysis (Figure 6F) revealed that L929 treated with dynamic RAW264.7‐CM (1:1) exhibited significantly accelerated wound closure compared to those treated with static RAW264.7‐CM (1:1). At 24 h post‐scratch, the dynamic CM group achieved 95% wound closure, versus 84% in the static CM group (*P < 0.001*), confirming that mechanically regulated macrophage paracrine factors enhance fibroblast migratory capacity.

Having established the migration and proliferation effect of macrophage secretions on fibroblasts, we hypothesized the existence of a reciprocal signalling loop. As shown in Figure 6G, Conditioned media from L929 fibroblasts, particularly from those subjected to prior mechanical stimulation (Dynamic L929‐CM), significantly upregulated the expression of both the canonical M2 marker CD206 and the key pro‐fibrotic cytokine TGF‐β1 in RAW264.7 macrophages, compared to media from static fibroblasts (Static L929‐CM). At the same time, we discovered that CM from mechanically stimulated macrophages (Dynamic RAW264.7‐CM) potently induced a pro‐adhesive and pro‐fibrotic state in L929 fibroblasts. Specifically, treatment with Dynamic RAW264.7‐CM led to a significant upregulation of FN1, a crucial extracellular matrix glycoprotein involved in cell adhesion and migration. Crucially, this was accompanied by the activation of the canonical TGF‐β1 signalling pathway. Based on the conclusions drawn from previous work, which showed that substrates with different mechanical properties can induce high expression of TGF‐β1 in macrophages and promote fibroblast proliferation [25], we hypothesized that shear force would induce both macrophages and fibroblasts to secrete more TGF‐β1. This, in turn, would promote fibroblast adhesion, the expression of fibrotic proteins, and M2 polarization of macrophages. We added exogenous TGF‐β1 to the cultures of both fibroblasts and macrophages. The results showed that following TGF‐β1 treatment, the M2 polarization‐related proteins Arg1 and CD206 in macrophages were highly expressed, whereas iNOS synthesis was suppressed. Concurrently, a significant increase in the expression of FN1 was observed in fibroblasts (Figure 6H). Therefore, in the microenvironment of soft tissue remodelling around the PEEK chest wall implant, macrophages and fibroblasts form a pro‐fibrotic positive feedback loop via a TGF‐β1‐dependent paracrine signalling pathway.

### 2.7 Chest PEEK Implants Promote Integration of Surrounding Soft Tissue Under Respiratory Alternating Shear Force

To validate the mechanism of soft tissue remodelling around chest wall PEEK implants under respiratory alternating forces in vivo, we established a comparative mouse subcutaneous implantation model. 6‐week‐old male BALB/cJGpt mice were used, with sterile PEEK implants placed in both the chest (dynamic site) and head (static site) of the same mouse. The implants, along with the surrounding soft tissues, were completely harvested at 1 and 2 weeks post‐operation for histological and mechanical analysis (Figure 7A). Masson's trichrome staining revealed extensive blue‐stained collagen fibre deposition around the chest implants, whereas minimal collagen deposition was observed around the head implants. Statistical analysis of the collagen fibre ratio in the soft tissue adjacent to the implant interface demonstrated that the collagen fibre content around the chest implants was significantly higher than that around the head implants (*P < 0.05*) (Figure 7B), indicating enhanced ECM deposition driven by dynamic mechanical stimulation. H&E staining showed that a structurally dense and cell‐rich fibrous layer had formed around the chest implants, with its thickness significantly greater than the loose connective tissue layer formed around the head implants (Figure 7C). Such mechanical stimulation drove a dynamic process of soft tissue integration at the implant periphery.

**FIGURE 7:**
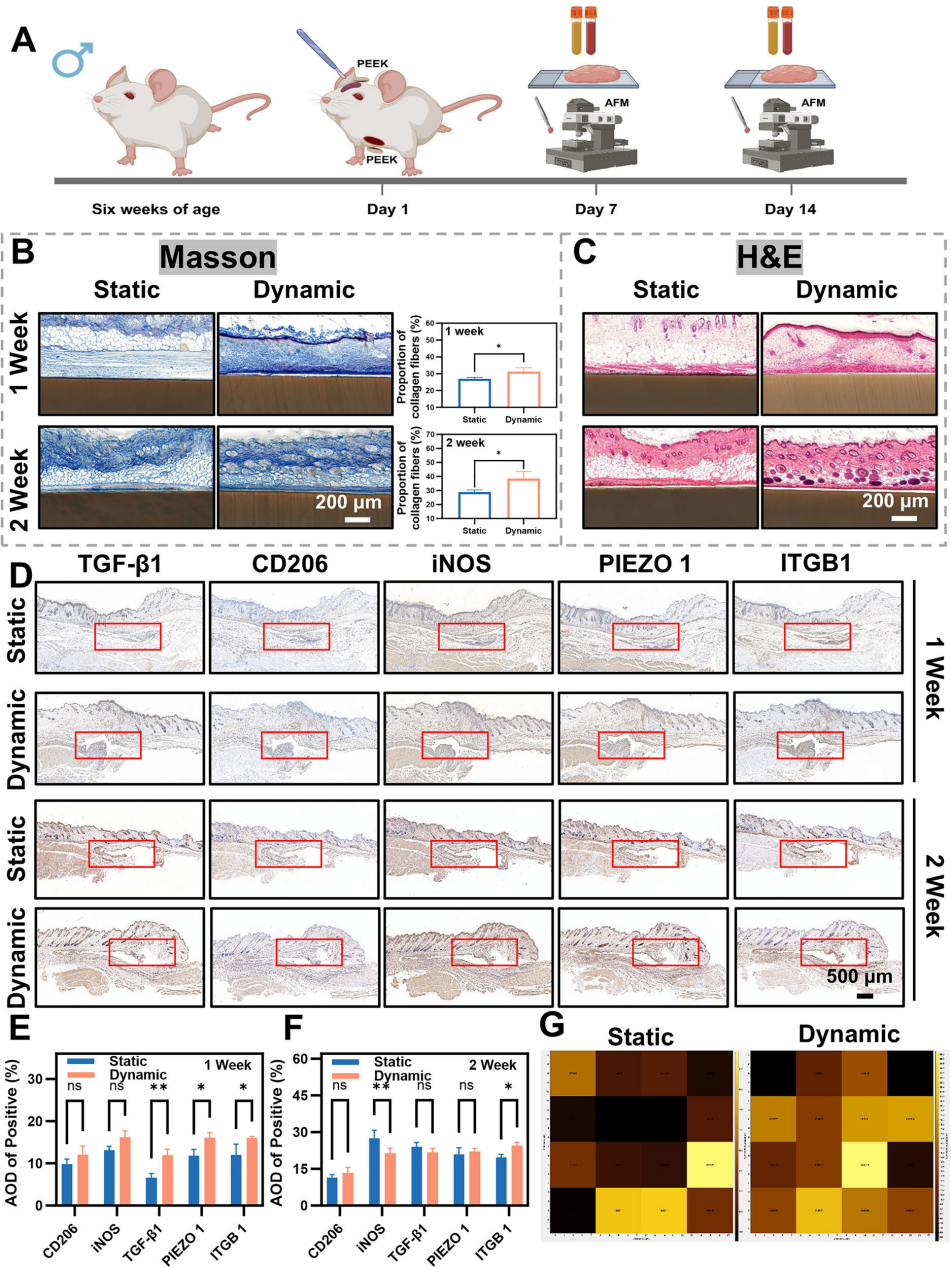
Chest wall PEEK implants promote soft tissue integration under dynamic respiratory mechanical cues in mice. (A) Schematic of the in vivo implantation experiment. (B) Masson's trichrome staining and (C) H&E staining of peri‐implant soft tissue(scale bar: 200 µm). (D) Immunohistochemical analysis of PIEZO1, TGF‐β1, ITGB1, iNOS and CD206 protein in soft tissues of different PEEK sample groups(scale bar: 500 µm). Quantitative analysis of immunohistochemical staining for PIEZO1, TGF‐β1, ITGB1, iNOS and CD206 presented after (E) 1 week and (F) 2 weeks. (G) AFM nanomechanical mapping. Shown are mean values ± SD (n = 3 independent experiments, each with 3 technical replicates), ^*^ *P* < 0.05, ^**^ *P* < 0.01, and ^***^ *P* < 0.001, ns, no significant difference. An unpaired t‐test was used in (B), and 2‐way ANOVA was used in (E)‐(F).

Subsequently, to elucidate the molecular mechanisms by which dynamic mechanical stimulation initiates tissue remodelling at the histological level, we assessed the differential expression of key mechanoreceptors as well as inflammation and fibrosis‐related proteins. Immunohistochemical analysis revealed that during the first week post‐implantation, the expression of the mechanosensitive ion channel PIEZO1, the core profibrotic factor TGF‐β1, and the adhesion protein ITGB1 was coordinately upregulated (Figure 7D). Notably, at this early stage, the expression of the classic pro‐inflammatory protein inducible nitric oxide synthase (iNOS) and the  anti‐inflammatory protein CD206 showed no significant differences between the chest and head implantation sites, suggesting that the initial foreign body reaction was similar in both mechanical environments (Figure 7E). By the second week post‐implantation, iNOS expression was significantly upregulated in the tissues surrounding the head implants. This indicates that the lack of mechanical stimulation leads to chronic inflammation in the tissue (Figure 7F). Biomechanical characterization via AFM further validated the functional consequences of mechanical stimulation. The Young's modulus of newly formed peri‐implant tissue was significantly higher in the chest group than in the head group (Figure 7G and Figure S12), providing direct mechanical evidence that dynamic stimulation induces dense collagen deposition and tissue stiffening.

To ensure that the observed local tissue responses were not associated with systemic toxicity, we conducted a histopathological examination of major organs two weeks after implantation. H&E staining of the heart, liver, spleen, lungs, and kidneys from mice with PEEK implants revealed normal tissue architecture and cellular morphology in all examined organs. This indicates that the PEEK implants exhibited good biocompatibility under the experimental conditions without inducing acute systemic toxic reactions (Figure S13A). Further serum biochemical analysis shows that key indicators of hepatic and renal function in both non‐implanted mice and PEEK‐implanted mice remained within normal reference ranges, with no statistically significant differences between the groups. This provides additional confirmation at the systemic metabolic level of the biosafety of PEEK material (Figure S13B).

## 3 Discussion

Retrospective clinical data have consistently shown that the risk of infection and soft‐tissue exposure after chest wall PEEK implantation is significantly lower than that in craniomaxillofacial defect repair [38, 39, 40]. While regional blood supply, soft tissue stiffness, microbial flora, and extracellular matrix composition have all been considered, the mechanical microenvironment intrinsic to each anatomical site has received little attention. To address this gap, we used a biomimetic mechanical culture system and animal models to show that respiratory shear stress transmits mechanical signals into pro‐reparative cellular responses. In fibroblasts, this occurs through the PIEZO1/ITGB1/YAP1 axis; in macrophages, through the PIEZO1‐STAT6/NF‐κB pathway. Within a defined mechanical threshold, cyclic shear force simultaneously promotes fibroblast activation, macrophage polarization, and paracrine crosstalk between the two cell types, ultimately generating a structurally stable soft tissue interface around the implant. Results from in vitro biomimetic culture were consistent with those from in vivo murine models, supporting the view that mechanical cues arising from physiological motion actively facilitate implant integration rather than merely accompanying the process. At the molecular pathway level, this finding offers an explanation for the differing outcomes of PEEK implants across anatomical sites. It also challenges the conventional development paradigm that focuses solely on chemical surface modification, suggesting that harnessing endogenous mechanical signaling may offer a viable alternative for improving soft tissue integration around implants.

This study revealed that simulated respiratory cyclic shear force enhanced the proliferation, adhesion, and migration of L929 cells on PEEK surfaces. Utilizing DNA tension probes, we identified 12 pN as the critical tension threshold for integrin β1 activation. Under static conditions, cellular anchoring traction forces were insufficient to reach this threshold, resulting in weak PIEZO1‐mediated calcium signaling, sustained suppression of the ITGB1/YAP1 pathway, and inadequate synthesis of collagen and fibronectin. In contrast, cyclic shear force continuously and stably elevated intracellular tension, activated the calcium signaling cascade, induced TGF‐β1 autocrine positive feedback, and persistently drove the ordered remodeling of the extracellular matrix. Numerous studies have established PIEZO1 as a mechanosensory [41, 42], consistent with the pathway results presented herein. However, previous integrin tension quantification systems were predominantly constructed on hydrophilic hydrogels or smooth glass substrates, rendering them incompatible with the cell anchoring deficiencies inherent to the hydrophobic nature of medical‐grade polymers [43, 44]. Prior efforts to improve PEEK biocompatibility have predominantly focused on hydrophilic modifications such as amination and hydroxylation [45, 46], which transiently enhance protein adsorption yet fail to provide sustained dynamic mechanical input. Consequently, cellular activity declines shortly after implantation. Both strategies must be considered synergistically to achieve long‐term maintenance of the reparative microenvironment at the implant interface.

Statically cultured fibroblasts generated traction forces below 12 pN, insufficient to activate tension probe fluorescence signals, whereas physiological shear force stably maintained cellular tension within this activation range. Existing studies have established the tension‐dependent nature of integrin activation [47, 48], but due to the aromatic ring structure of PEEK [49], which confers strong hydrophobicity and substantially attenuates cellular adhesion, statically cultured fibroblasts cannot be affected by traction forces above the threshold required for activating downstream fibrogenic pathways, which hinders the formulation of quantitative standards for clinical PEEK materials. Respiratory shearforce, however, compensates for this mechanical deficiency and sustains the maturation and activation window of integrins. Thus, we used physiological shear force to stably maintain cellular tension within this activation range, representing the core innovation that distinguishes this study from prior mechanosensing investigations.

The macrophage response furnishes an additional dimension for understanding immune regulation during the integration process. Short‐term shear force promoted RAW264.7 cells toward the M1 phenotype, whereas sustained mechanical intervention for 12 h progressively directed cellular polarization toward the reparative M2 phenotype. This temporal regulation was governed by competitive regulation between the NF‐κB and STAT6 pathways downstream of PIEZO1. Previous studies have independently elucidated NF‐κB‐mediated acute inflammation and STAT6‐driven tissue repair [50, 51], yet few have demonstrated that cyclic shear force can temporally switch the activity of these two pathways. Short‐term mechanical perturbation constitutes a foreign‐body stress signal that preferentially activates the NF‐κB inflammatory program, whereas mechanical stimulation that persists for 12 h activates the STAT6 signaling pathway downstream of PIEZO1, which becomes significantly upregulated. Activated STAT6 suppresses NF‐κB transcriptional activity through cross‐regulation between transcription factors, thereby progressively resolving chronic inflammatory damage. In contrast to static material studies, this study demonstrates that mechanical intervention can actively remodel the local immune microenvironment at the implantation site through temporal regulation, compensating for the limitation of single‐modality material modifications in achieving sustained inflammatory control.

Fibroblast‐mediated matrix remodeling and macrophage immune regulation are not two independent processes. They constitute an integrated repair network through a shear force‐dependent, bidirectional TGF‐β1 paracrine loop. The majority of in vitro co‐culture studies require exogenous supplementation with recombinant TGF‐β1 to induce reparative phenotypes [52, 53], thereby departing from the logic of physiological regulation in vivo. Under static conditions, the scarcity of extracellular matrix precludes the release of latent TGF‐β1, resulting in the interruption of intercellular signal transmission. We demonstrated that shear force induces substantial collagen deposition, and matrix‐bound latent TGF‐β1 is mechanically activated and released. Meanwhile, fibroblast‐secreted factors subsequently drive macrophage M2 polarization, and then polarized macrophages reciprocally secrete cytokines that promote fibroblast migration and collagen synthesis, establishing a self‐sustaining reparative microenvironment. This interactive mechanism addresses the limitations of prior single‐cell in vitro experiments and more closely approximates the authentic physiological state of multi‐cellular collaborative participation in implant repair in vivo.

Moreover, bilateral subcutaneous implantation in the same mouse demonstrated that the dynamic chest site exhibited more abundant collagen deposition and significantly attenuated chronic inflammatory infiltration compared with the static craniomaxillofacial site. Current clinical PEEK prostheses predominantly employ uniform surface‐modification strategies, thereby overlooking the mechanical environmental heterogeneity across distinct anatomical sites and, consequently, constraining their applicability. The craniomaxillofacial region lacks endogenous cyclic shear force; relying solely on material modification proves insufficient for long‐term control of foreign‐body inflammatory responses. In contrast, the thoracic wall can leverage the intrinsic mechanical signals generated by respiration, requiring only optimization of the interfacial architecture to lower the 12 pN force activation threshold, thereby substantially enhancing soft tissue integration outcomes. This site‐specific differential design concept is not confined to PEEK prostheses for thoracic wall defects, but can be extended to vascular stents, abdominal wall soft tissue patches, dynamic spinal fusion devices, and other implantable instruments subjected to long‐term cyclic mechanical loading, thereby furnishing a novel paradigm of mechanoregulation for the development of next‐generation biomaterials.

Despite these important findings, there is still room for further research based on our study. First, although the murine subcutaneous implantation model established a correlation between mechanical stimulation and soft tissue repair, it remains insufficient to substantiate a causal relationship, given that the chest wall and craniomaxillofacial sites exhibit inherent biological disparities across multiple dimensions, including angiogenesis, local ECM composition, tissue thickness, mechanical restriction, and immune composition. Second, the subcutaneous implantation microenvironment differs to some extent from the in situ physiological environment of the thoracic cavity. Subcutaneous oxygen concentration, efficiency of humoral circulation, and the amplitude of respiratory micromotion transmission all deviate from those of authentic chest wall soft tissue, thereby partially attenuating the transduction efficiency of mechanical signals into intracellular pathways and influencing cellular responsiveness. Third, all in vitro mechanistic validations were conducted using murine‐derived immortalized L929 and RAW264.7 cell lines, whose mechanosensory properties and cytokine secretion patterns may differ from those of human primary chest wall cells. To address these limitations, future investigations could employ thoracic wall in situ animal models, concurrently isolate thoracic wall tissue from patients to obtain human primary fibroblasts and macrophages to replicate core experiments, and verify pathway conservation in humans. Additionally, a prosthesis rigid‐fixation control group could be incorporated to selectively eliminate the micromotion variable, thereby further substantiating the causal association between respiratory shear stress and soft‐tissue integration.

## 4 Conclusion

In this study, a dynamic cell culture system was constructed to simulate human respiratory movement. Through finite element analysis, it is proven that the dynamic culture system we developed can effectively reproduce the biomechanical microenvironment generated by human respiratory movement at the junction of the implant and tissue. The results showed that physiological‐scale respiratory alternating shear stress mediated integration of soft tissue around PEEK by activating the PIEZO1/ITGB1/YAP1 signalling axis in fibroblasts and the STAT6/p‐STAT6 pathway in macrophages. The force‐sensitive reaction of integrin β1 is triggered within a precise 12 pN molecular tension range. Further results indicated that L929 fibroblasts and RAW264.7 macrophages orchestrate reciprocal crosstalk through TGF‐β1 paracrine signalling, forming a positive feedback regulatory loop. Animal studies also demonstrated better soft tissue integration around chest wall PEEK implants than around craniomaxillofacial PEEK implants.

This finding establishes a comprehensive approach for a dynamic mechanical microenvironment that regulates tissue integration from the multi‐scale perspective of mechanical loading, molecular sensing and cell interaction. Our mechanobiology‐oriented findings expand the mechanistic understanding of site‐dependent PEEK implant performance and provide a theoretical basis for the site‐specific design of PEEK implants for different anatomical locations.

## 5 Experimental Section

### 5.1 Materials

CO_2_ production gas package was purchased from Mitsubishi Chemical Group. A controllable temperature insulation package was purchased from Wantian Fukang Electronic Factory. PEEK powder was obtained from Victrex plc. NCTC clone 929(L929) and RAW 264.7 were purchased from the Institute of Biochemistry and Cell Biology, Shanghai Institute of Life Sciences. 0.25% Trypsin Solution (with EDTA, phenol red, dissolved in D‐Hank's), MEM (with NEAA) and DMEM (High glucose) were purchased from Procell System. Fetal bovine serum (FBS) was obtained from Biological Industries. Phosphate Buffered Saline×1 was purchased from Service Bio Technology Company. Penicillin Streptomycin Solution, Fluo‐4 AM, and CCK‐8 assay kit were purchased from New Cell & Molecular Biotech; a Live/Dead cell imaging kit was purchased from Beyotime Biotechnology Company; FITC Phalloidine was obtained from Yeasen Biotech Company; anti‐fluorescence attenuation sealing agent and DAPI were purchased from Boster Biological Technology Company. Hexamethyl disilylamine was obtained from Psaitong Biotechnology Company, and HAuCl_4_·xH_2_O was purchased from Aladdin.

### 5.2 Construction of Cell Dynamic Culture System

Establish the concentration ratios of CO_2_ and O_2_ required for cell culture, set a suitable temperature (37 °C), and achieve controllable, adjustable culture conditions. The culture system consists mainly of three parts: a 2D shaking device, a constant‐temperature (37°C) control device, and a carbon dioxide (5%) constant‐pressure device.

### 5.3 Characterization of PEEK Discs

The composition of PEEK was characterized by a mapping (Apreo HiVac, FEI Company, USA) and an X‐ray photoelectron spectrometer (XPS, PHI VersaProbe 4). The surface morphology of PEEK was observed by an atomic force microscope (AFM) and a field emission scanning electron microscope (SEM) (Apreo HiVac, FEI Company, USA). The surface hardness of PEEK was characterized by Bruker HysitronTi Premier (*2 × 2/*Ti Premier). The surface wettability of PEEK, PEEK‐APTES, PEEK‐APTES‐Au, and PEEK‐K(N_3_)GRGDNP samples was evaluated using a fully automated water contact angle measuring instrument.

### 5.4 Cell Experiments

Mouse‐derived L929 and RAW 264.7 were chosen for studies in vitro. L929 cells were seeded in cell culture flasks and cultured with MEM(with NEAA) containing 10% FBS and 1% penicillin and streptomycin, maintained at 37 °C in a humidified atmosphere with 5% CO_2_. Similarly, RAW 264.7 cells were seeded in cell culture flasks and cultured with DMEM (High glucose) containing 10% FBS and 1% penicillin and streptomycin.

### 5.5 Finite Element Analysis (FEA)

To investigate the motion and stress distribution of cells within a dynamic cell culture system, numerical simulations were conducted using COMSOL Multiphysics finite element software. The physical model coupled the laminar‐flow interface, the solid‐mechanics interface, and the arbitrary Lagrangian‐Eulerian (ALE) moving mesh method. Based on continuum mechanics, the solid mechanics module solved for displacement, stress, and strain within the cell structure using the momentum conservation Equation (1), kinematic relations Equation (2), and constitutive laws for linear elastic materials Equation (3).

(1) $$\rho \;\frac{\partial^{2}u}{\partial t^{2}}=\nabla \cdot S+F_{V}$$

(2) $$\varepsilon =\frac{1}{2}\;\left(\nabla u+{\left(\nabla u\right)}^{T}\right)$$

(3) $$C=C\left(E,\nu \right)$$

The fluid dynamics of the surrounding medium were described by the continuity Equations (4 and 5) and Navier‐Stokes Equation (6) for incompressible flow. Two geometric configurations were established to model suspended and adherent cells within the dynamic cell culture system.

(4) $$\frac{\partial \rho }{\partial t}+\nabla \cdot \;\left(\rho u\right)=0$$

(5) ∇·u=0

(6) $$\frac{\partial \left(\rho u\right)}{\partial t}+\nabla \cdot \;\left(\rho uu\right)=\nabla \cdot \left[\mu \left(\nabla u+\nabla u^{T}\right)\right]-\nabla p+F$$

*ρ*: material density; u: displacement vector; S: The first kind of Piola‐Kirchhoff stress tensor; F_V_: Volumetric force per unit volume; ∇: divergence operator; C: elastic stiffness modulus; E: young's modulus; ν: poisson ratio.

### 5.6 Immunoblot Analysis

L929 cells were seeded on PEEK surfaces at a density of 5 × 10^5^ cells per well and cultured under both dynamic and static conditions for 6, 12, and 24 h. After culturing, the cells were detached from the PEEK surfaces using trypsin, collected, and washed with PBS. The collected cells were then treated with cell lysis buffer for 20 min, after which the samples were harvested. From each sample, 5 µL was taken for protein quantification by measuring absorbance at 562 nm. The remaining sample was mixed with protein loading buffer and boiled for 5 min. The proteins were separated by gel electrophoresis and subsequently transferred to a PVDF membrane for immunoblotting. The membrane was blocked at room temperature for 2 h with a blocking buffer containing 1% skimmed milk in TBST. Following blocking, the membrane was incubated with the primary antibody overnight at 4 °C. After incubation, the membrane was washed three times with TBST and then incubated with the secondary antibody at room temperature for 1 h. Secondary antibodies used at 1:5000 dilutions in blocking solutions were anti‐rabbit (1:5000, EK020, Xi'an Zhuangzhi Biotechnology Co., Ltd., China) and anti‐mouse (1:5000, EK010, Xi'an Zhuangzhi Biotechnology Co., Ltd., China). Primary antibodies used were rabbit anti‐Piezo1 (1:750; A23380, Wuhan, ABclonal), rabbit anti‐Fibronectin (1:750; A25907, Wuhan, ABclonal), mouse anti‐Smad2/3(1:500; sc‐133098, Santa Cruz Biotechnology, Inc.), rabbit anti‐TGF‐β1 (1:2500, 21898‐1‐AP, Proteintech Group), rabbit anti‐α‐SMA (1:6000, 14395‐1‐AP, Proteintech Group), rabbit anti‐YAP1 (1:5000, 13584‐1‐AP, Proteintech Group), anti‐ITGB1(1:100, 34971T, Cell Signaling Technology, Inc).

The Western blot experiment for macrophages was performed consistently with the procedures described above. Primary antibodies used were rabbit anti‐CD206, (1:2500, ET1702‐04, HUABIO), mouse anti‐iNOS(1:1000, TA0199S, Abmart), mouse anti‐IL‐10(1:500, 2G101H7, MedChemExpress LLC., USA), Rabbit anti‐NF‐*κ*B(1:1000, T55034, Abmart), rabbit anti‐p‐NF‐*κ*B (1:1000, TP56372, Abmart), mouse anti‐p‐STAT6 (1:1000, F0555, Selleck), mouse anti‐STAT6 (1:1000, F0412, Selleck).

### 5.7 Real‐Time Polymerase Chain Reaction Analysis

For Real‐Time Polymerase Chain Reaction Analysis, mRNA was extracted by the M5Universal RNA Mini Kit (MF036‐01, Mei5Biotechnology, Co., Ltd) according to the manufacturer's instructions. RNA (500 ng) was reverse transcribed with the MightyScript First Strand cDNA Synthesis Master Mix(B639251‐0100, Sangon Biotech (Shanghai) Co., Ltd.). PCR amplification was performed in triplicate by SGExcel FastSYBR Mixture(B532955, Sangon Biotech (Shanghai) Co., Ltd., China) using a qPCR instrument (MX3500P, Agilent, USA) at 95 °C for 3 min, 38 cycles at 95°C for 15 s, at 60°C annealing temperature for 20 s followed by the melt curve. All samples were normalized using β‐actin.

*β‐actin*(GTGACGTTGACATCCGTAAAGA; GCCGGACTCATCGTACTCC), *TGF‐β1*(CCACCTCAAGACCATCGAC; CTGGCGAGCCTTAGTTTGGAC), *α‐SMA*(CCCAGACATCAGGGAGTAATGG; TCTATCGGATACTTCAGCGTCA), *PIEZO1* (CCTGTTACGCTTCAATGCTCT; GTGTAGGCATATCTGAAAGGCAA), ITGB 1(ATGCCAAATCTTGCGGAGAAT; TTTGCTGCGATTGGTGACATT).

*IL10*(GCTCTTACTGACTGGCATGAG; CGCAGCTCTAGGAGCATGTG), *iNOS*(ACATCGACCCGTCCACAGTAT; CAGAGGGGTAGGCTTGTCTC), *TNF‐α*(CTGAACTTCGGGGTGATCGG; GGCTTGTCACTCGAATTTTGAGA), *MGL 1*(CAATGTGGTTAGTTGGATCGGC; CCCAGTTCTTAAAGCCTTTCTCA).

### 5.8 DNA Tension Probe Preparation

The molecular tension probes used in the present study were modified based on previously reported methodologies [34]. Three types of DNA probes with distinct force thresholds were designed, including an unzipping mode (12 pN), intermediate mode (33 pN), and shear mode (56 pN), with their sequences as follows:

Unzipping 12 pN:

5′‐/DBCO/CACAGCACGGAGGCACGACAC/BHQ2/‐3′

Intermediate 33 pN:

5′‐CACAGCA/iDBCOdT/CGGAGGCACGACAC/BHQ2/‐3′

Shear 56 pN:

5′‐/BHQ2/CACAGCACGGAGGCACGACAC/DBCO/‐3′

All DNA oligonucleotides were purchased from Sangon Biotech (Shanghai, China) and used without further purification.

Conjugation of the azide‐modified cyclic peptide to the DNA oligonucleotide was achieved via copper‐free azide–alkyne cycloaddition (SPAAC). Briefly, K(N_3_)GRGDNP (azide‐modified cyclic peptide, MCE, Shanghai, China) and DBCO‐modified DNA oligonucleotide (Sangon Biotech) were mixed at a molar ratio of 2:1 in 1×TAE‐Mg^2^^+^ buffer (containing 10 mM MgCl_2_, pH 8.0). The reaction mixture was incubated overnight at 4 °C to allow covalent conjugation between the cyclic peptide and DNA strand. The resulting product (K(N_3_)GRGDNP‐oligonucleotide‐BHQ2) was used directly in subsequent DNA origami assembly experiments without additional purification.

DNA origami nanostructures integrated with molecular tension probes were assembled as follows: The scaffold single‐stranded DNA (ssDNA) was extracted from bacteriophages according to a previously reported protocol [54]. Staple strands were designed from the bacteriophage genome sequence to facilitate folding of the scaffold ssDNA into the desired nanostructure. Three functional DNA strands—K(N_3_)GRGDNP‐oligonucleotide‐BHQ2 (targeting integrin β1), thiol‐modified oligonucleotides (for adsorption onto gold nanoparticles (AuNPs) decorated on PEEK surfaces), and Cy3‐modified strands (component of the fluorescence quenching pair)—were hybridized to specific positions on the scaffold ssDNA via sequence‐complementary pairing (Figure S8).

For DNA origami assembly, the scaffold ssDNA, K(N_3_)GRGDNP‐oligonucleotide‐BHQ2, Cy3‐modified strands, and staple strands (including all modified oligonucleotides) were mixed in 1×TAE‐Mg^2^^+^ buffer at a molar ratio of 1:2:2.5:10. The reaction mixture was placed in a thermal cycler (Bio‐Rad, USA) and annealed using the following program to enable specific hybridization and structural folding: gradual cooling from 85 °C to 65 °C over 21 min, followed by gradual cooling from 64 °C to 45 °C over 1 h and 40 min, then gradual cooling from 44 °C to 30 °C over 15 min, and finally sequential incubation at 25 °C for 5 min, 20 °C for 5 min, and 15 °C for 5 min. After annealing, the resulting DNA origami nanostructures integrated with molecular tension probes were directly dropped onto AuNPs‐decorated PEEK surfaces and allowed to adsorb spontaneously at room temperature for 1 h to complete probe immobilization.

### 5.9 DNA Tension Probe Modification on PEEK Surfaces

The PEEK substrate was ultrasonically cleaned three times (15 min each) in anhydrous ethanol and deionized water, followed by drying in an oven at 60 °C. The dried PEEK substrate was placed in a plasma chamber and treated with air plasma for 10 min under the conditions of a gas flow rate of 35 sccm and a pressure of 200 mTorr. The PEEK substrate was treated by immersion in a 1% (v/v) APTES (3‐Aminopropyl trimethoxysilane) solution in ethanol. After silanization, the PEEK substrate was rinsed twice with ethanol to remove unbound APTES. Subsequently, the surface was dried under a stream of nitrogen gas. The substrate was then immersed in 5 mL of HAuCl_4_ solution (25 mM) and incubated at room temperature for 4 h to allow for the in‐situ formation and attachment of AuNPs. Finally, the sample was thoroughly rinsed with ultrapure water to remove any unbound AuNPs. The DNA tension probe was dropped onto the PEEK‐APTES‐Au surface and incubated overnight at 4 °C.

### 5.10 M1 and M2 Polarization of Macrophages

To induce M1 phenotype, M0 macrophages were stimulated with 2.5 µg/mL lipopolysaccharide (LPS) (from E. coli, serotype O55:B5, Targetmol, T11855)for 24 h. After stimulation, cells were harvested for subsequent analyses. To induce M2 phenotype, M0 macrophages were stimulated with 10 ng/mL IL‐10 (HY‐P70517, MedChemExpress LLC., USA) and 20 ng/mL IL‐4 (HY‐P70653, MedChemExpress LLC., USA)

### 5.11 In Vivo Animal Experiments

#### 5.11.1 Surgical Implantation

All animal experimental procedures were authorized by the Ethics and Welfare Committee of the Experimental Animal Center of the Fourth Military Medical University, approval number:241194. A total of 18 BALB/cJGpt mice(6 weeks old, male) were used for the in vivo soft tissue regeneration experiment. The mice underwent one week of preoperative adaptive housing. Isoflurane inhalation anesthesia was administered, followed by shaving of the chest and head areas. The skin was then disinfected with iodophor. The mice were placed on a sterile surgical drape. An approximately 0.7 cm incision was made on the chest and head. Using sterile instruments, the subcutaneous tissue was dissected, and PEEK was implanted into the subcutaneous tissue of the head and chest, respectively. Meanwhile, the blank group had only skin incisions followed by sutures for biosafety controls. Finally, absorbable sutures were used to close the wound. The mice were placed on a warming pad until they regained consciousness. Tissue samples containing the implants and surrounding soft tissue were collected at the first and second weeks post‐operation. These samples were fixed in 4% paraformaldehyde at room temperature for 24 h, followed by subsequent experiments and characterization. The fixed implant‐soft tissue composite samples were thoroughly rinsed with PBS buffer, followed by gradient dehydration of the samples. The resin‐embedded samples were then sectioned into approximately 20 µm thick, uniform slices using a hard tissue microtome. Subsequently, H&E and Masson staining were performed.

#### 5.11.2 Immunohistochemical Analysis

The harvested tissue and implant were separated and fixed in 4% paraformaldehyde for 24 h, followed by paraffin embedding. Slices were deparaffinized and rehydrated using a graded ethanol series. Antigen retrieval was then performed. Endogenous peroxidase activity was blocked with 3% hydrogen peroxide solution, and non‐specific binding sites were blocked by incubating with BSA blocking solution at room temperature for 30 min. Appropriately diluted primary antibodies (TGF‐β1, PIEZO1, ITGB1, CD206) were applied, and the slides were incubated overnight at 4 °C in a humidified chamber. Subsequently, secondary antibody was applied and incubated at room temperature for 30 min. Color development was performed using DAB chromogen, followed by counterstaining with hematoxylin. Finally, the slides were mounted with neutral resin mounting medium.

#### 5.11.3 Biocompatibility Assessment of PEEK

To evaluate the systemic toxicity of the implant and its effects on the visceral organs in mice, peripheral blood was collected from BALB/cJGpt mice at one and two weeks post‐operation via retro‐orbital venous plexus sampling into tubes. Subsequent whole blood cell count analysis and biochemical analysis were performed. The mice were then euthanized, and organs (heart, liver, spleen, lungs, kidneys) were collected for tissue sectioning and staining.

### 5.12 Statistical Analysis

Performed using GraphPad Prism 10.0. Data are presented as mean ± standard deviation (SD). Unless otherwise specified in the figure legends, all experiments were performed with at least three independent biological replicates (n ≥ 3), and data from each biological replicate were obtained from three technical replicates. Comparisons between two groups were analysed using a two‐tailed unpaired *t*‐test, while comparisons among multiple groups were assessed using one‐way or two‐way analysis of variance (ANOVA) followed by appropriate post hoc tests. Statistical significance was defined as *P* < 0.05(^*^), *P* < 0.01(^**^), *P* < 0.001(^***^), and *P* < 0.0001(^****^).

## Author Contributions

**Rou Huang**: methodology, Writing – original draft, conceptualization, data curation, investigation. **Xiao Liang**: investigation. **Xing Li**: investigation. **Yangfan Huo**: conceptualization, methodology, data curation, validation. **Ying Sun**: investigation. **Sida Liu**: conceptualization, methodology. **Hao Guo**: investigation, validation. **Jin Liu**: conceptualization, methodology, data curation. **Jiawei Xiu**: investigation. **Xiaolong Yan**: writing – review and editing, project administration. **Yuanquan Zhang**: validation. **Lijun Huang**: writing – review and editing, project administration, resources, writing – original draft. **Weiwei Wu**: writing – review and editing, resources. **Lei Wang**: writing – review and editing, project administration, resources, visualization, writing – original draft. **Minghai Ma**: investigation, validation.

## Ethics Statement

This study was approved by the Ethics and Welfare Committee of the Experimental Animal Center of the Fourth Military Medical University (Approval No: 241194).

## Conflicts of Interest

The authors declare no conflict of interest.

## Supporting information




**Supporting File**: advs77118‐sup‐0001‐SuppMat1.docx.

## Data Availability

The data that support the findings of this study are available from the corresponding author upon reasonable request. The raw RNA sequencing data have been deposited in the Sequence Read Archive (SRA) database with accession numbers SRR39452411, SRR39452410, SRR39452409, SRR39452406, SRR39452407 and SRR39452408.
