# Supplementary material for: Alternating Shear Force of Respiration Regulates Cell Interactions of Fibroblasts and Macrophages to Promote Soft Tissue Integration of Chest Wall Polyetheretherketone Implants
Source: Adv Sci (Weinh). 2026 Aug 17;13(62):e77118. doi: 10.1002/advs.77118 (PMC13479593; doi:10.1002/advs.77118)
Supplement: Supplementary file 1 [file ADVS-13-e77118-s001.docx]

Supporting Information

Alternating Shear Force of Respiration Regulates Cell Interactions of Fibroblasts and Macrophages to Promote Soft Tissue Integration of Polyetheretherketone Implants

Rou Huang^1,2#^, Yangfan Huo^1#^, Jin Liu^1#^, Sida Liu^1^, Minghai Ma^1^, Hao Guo^1^, Yuanquan Zhang^1^, Xing Li^1^, Xiao Liang^1^, Jiawei Xiu^1^, Ying Sun^1^, Lijun Huang^1*^, Xiaolong Yan^1*^, Weiwei Wu^3*^, Lei Wang^1*^

**Figure S1**. Cell viability of L929 cells cultured under dynamic (different mechanical force stimuli, various time points) and static conditions was measured by CCK-8.

**Figure S2. (**A). Colony formation images of L929 cells after shear stress stimulation. (B). Quantitative analysis of colony numbers in panel (A).

**Figure S3.** Representative images of suspended cells in the culture supernatant of L929 after 12 h incubation under dynamic and static conditions (optical microscope).

**Figure S4.** Gene expression of L929 were cultured under dynamic (40 rpm) and static conditions for 12 h.

**Figure S5.** Schematic illustration of the synthesis procedure of AuNPs.

**Figure S6. (**A). HAADF-STEM image of single Au-DNA origami particle (left, scale bar=20 nm), together with corresponding EDS elemental mapping of Au, S, and P, confirming the uniform co-distribution of Au core and DNA origami shell elements within the hybrid nanostructure. (B). AFM images of PEEK, PEEK-APTES, PEEK-APTES-Au and DNA origami-K(N_3_)GRGDNP.

**Figure S7**. Denaturing PAGE gel images of DBCO-modified DNA strand.

**Figure S8.** Schematic diagram of DNA origami-based molecular tension probe.

**Figure S9**. Schematic diagram of DNA origami structure.

**Figure S10**. (A). Western blot analysis of polarization-related signalling proteins in RAW264.7 macrophages under four culture conditions: static culture without stimulation, dynamic respiratory shear stress, static culture with LPS treatment (standard M1 positive control), and static culture with combined IL-10+IL-4 treatment (standard M2 positive control). (B). Immunofluorescence staining of M2 surface marker CD206 and M1 marker iNOS in control, IL-10+IL-4-treated and LPS-treated RAW264.7 macrophages. Cell nuclei were stained with DAPI. Scale bar: 40 μm. (C). Scanning electron microscopy (SEM) showing characteristic cellular morphology of static, LPS-induced M1, and IL-10+IL-4-induced M2 macrophages. Scale bar: 20 μm.

**Figure S11**. b. PIEZO1 knockdown activated STAT6 expression, whereas it suppressed NF-κB protein levels.

**Figure S12**. Statistical analysis of AFM nanomechanical mapping

**Figure S13**. (A). Representative H&E staining images of heart, liver, spleen, lung, and kidney from mice with PEEK implantation versus control mice without implantation. (B). Hematological and serum biochemical parameters detected in mice two weeks after PEEK implantation. Assessed parameters included white blood cell count (WBC), lymphocyte count (Lymph#), monocyte count (Mon#), granulocyte count (Gran#), red blood cell count (RBC), hemoglobin (HGB), platelet count (PLT), and hematocrit (HCT)
